# Qualitative and Quantitative Analysis of Major Triterpenoids in *Alismatis Rhizoma* by High Performance Liquid Chromatography/Diode-Array Detector/Quadrupole-Time-of-Flight Mass Spectrometry and Ultra-Performance Liquid Chromatography/Triple Quadrupole Mass Spectrometry

**DOI:** 10.3390/molecules200813958

**Published:** 2015-07-31

**Authors:** Wanli Zhao, Xiaoqiang Huang, Xiaoyan Li, Fangfang Zhang, Sainan Chen, Miao Ye, Mingqing Huang, Wen Xu, Shuisheng Wu

**Affiliations:** 1 College of Pharmacy, Fujian University of Traditional Chinese Medicine, Fuzhou 350122, China; E-Mails: zhaowanlitcm@126.com (W.Z.); 15880081619@163.com (X.H.); lixy8556@163.com (X.L.); rdfxzff@163.com (F.Z.); 18950490357@163.com (S.C.); ymfjtcm@163.com (M.Y.); huangmingqing3413@gmail.com (M.H.); 2 Institute of Nanostructured Functional materials, Huanghe Science and Technology College, Zhengzhou 450006, China

**Keywords:** *Alismatis Rhizoma*, *Alisma orientale* (Sam.) Juzep., quality control, QTOF mass spectrometry, QqQ mass spectrometry

## Abstract

*Alismatis Rhizoma* (AMR) is a well-known natural medicine with a long history in Chinese medicine and has been commonly used for treating a wide range of ailments related to dysuria, edema, nephropathy, hyperlipidaemia, diabetes, inflammation as well as tumors in clinical applications. Most beneficial effects of AMR are attributed to the presence of protostane terpenoids, the major active ingredients of *Alismatis Rhizoma* (AMR). In this study, a systematic high performance liquid chromatography/diode-array detector/quadrupole-time-of-flight mass spectrometry (HPLC-DAD-Q-TOF MS) and ultra-performance liquid chromatography/triple quadrupole mass spectrometry (UPLC-QqQ MS) method was developed for qualitative and quantitative analyses of the major AMR triterpenoids. First, a total of 25 triterpenoid components, including 24 known compounds and one new compound were identified by comparison with UV spectra, molecular ions and fragmentation behaviors of reference standards or the literature. Second, an efficient method was established for the rapid simultaneous determination of 14 representative triterpenoids by UPLC-QqQ MS. Forty-three batches of AMR were analyzed with linearity (*r*, 0.9980–0.9999), intra-day precision (RSD, 1.18%–3.79%), inter-day precision (RSD, 1.53%–3.96%), stability (RSD, 1.32%–3.97%), repeatability (RSD, 2.21%–4.25%), and recovery (98.11%–103.8%). These results indicated that new approaches combining HPLC-DAD-Q-TOF MS and UPLC-QqQ MS are applicable in the qualitative and quantitative analysis of AMR.

## 1. Introduction

*Alismatis Rhizoma* (AMR) are dried rhizomes of *Alisma orientale* (Sam.) Juzep., it is a well-known natural medicine with long history in Chinese medicine. As a traditional medicine in China, AMR is an important part of many prescriptions and has been commonly used for treating a wide range of ailments related to dysuria, edema, nephropathy, hyperlipidaemia, diabetes, inflammation as well as tumors in clinical applications [1,2,3,4,5]. Most beneficial effects of AMR are attributed to the presence of protostane terpenoids, which are relatively abundant in this preparation. Previous studies have indicated that triterpenoids have diverse biological activities such as hypolipidemic [1], anti-proliferative activities [6], antibacterial [7], antiplasmodial [8], antioxidative [9] and anti-inflammatory bioactivities [10].

Previously, multiple studies have reported the analysis of triterpenes in AMR by LC-UV, LC-ELSD and LC-MS [11,12,13]. However, they focused on characterizing the limited compounds, with shortcomings such as long analysis time and poor LOQs [11,12], sometimes providing only qualitative data [13]. To the best of our knowledge, few studies assessing the systematic chemical profile and quantification of AMR were reported. Therefore, it is of great significance to develop a method for qualitative and quantitative analyses of AMR chemical constituents, which would be beneficial to studies evaluating AMR efficacy and quality.

Recently, liquid chromatography-mass spectrometry (LC-MS) offers the possibility to obtain a more comprehensive chemical profile and quantitative by utilizing multiple ionization techniques and/or different ion modes [14,15,16]. LC coupled with the time-of-flight (TOF) mass spectrometry, it has the capability and advantage to produce exact mass measurements, which provides the elemental compositions of unknown peaks with high accuracy. It also provides data on accurate precursor and/or product ions with high accuracy (routinely below 5 ppm), which substantially enhances the metabolite characterization reliability [17,18]. Beside, simultaneous quantification of multi-components has been widely performed in the analysis of traditional Chinese medicine (TCM) [19]. The ultra-performance liquid chromatography (UPLC) method has become one of the most frequently applied approaches in the area of fast chromatographic separations. Moreover, triple quadrupole mass spectrometry (QqQ MS) has higher sensitivity than ELSD and UV detections, especially for the non-UV-absorbing such as triterpenoids present in AMR. UPLC coupled with triple quadrupole mass spectrometry (UPLC QqQ MS) with high sensitivity and effectiveness provides a reliable quantification of multi-components in TCM [20,21].

In this study, a systematic high performance liquid chromatography/diode-array detector/quadrupole-time-of-flight mass spectrometry (HPLC-DAD-Q-TOF MS) and ultra-performance liquid chromatography/triple quadrupole mass spectrometry (UPLC-QqQ MS) method was developed for qualitative and quantitative analyses of the major triterpenoids in AMR. First, 21 triterpenoid compounds were clearly identified by comparison with the reference standards, while four triterpenoids, including one new compound (16-oxo-11-deoxy-alisol A), were tentatively identified by comparison with the UV spectra, molecular ions, and fragmentation behaviors. Second, quantification of 14 representative compounds, including 16-oxo-alisol A 23-acetate, 16-oxo-alisol A 24-acetate, alisol C, alisol F, alisol C 23-acetate, alisol L, alisol F 24-acetate, alisol A, alisol A 23-acetate, alisol A 24-acetate, alisol G, alisol B, alisol B 23-acetate and 11-deoxy-alisol B, was carried out using the UPLC-QqQ MS method. This validated method was subsequently applied to characterize forty-three batches of AMR samples.

## 2. Results and Discussion

### 2.1. Optimization of Sample Preparation

Different extraction solvent systems (methanol-water solution and acetonitrile-water solution at 40%, 60%, 80% and 100%), procedures (refluxing, Soxhlet extraction and ultrasonication) and times (15, 30, 45 and 60 min) were evaluated. The optimal sample preparation was found to be 0.20 g powder, ultrasonicated for 30 min in 25 mL acetonitrile (Supplementary Figure S1).

### 2.2. Optimization of HPLC-DAD-Q-TOF MS Conditions

For qualitative analysis, in order to improve HPLC resolution and sensitivity, and shorten the analytical time, variables such as column type, column temperature, mobile phase, and flow rate can be optimized. We found that use of Ultimate XB ODS-C_18_ column (4.6 × 150 mm, 5 μm, Welch, Concord, MA, USA) results in improved peak capacity, stronger retention ability, and better resolution compared with other columns; therefore, it was selected for the study. Different mobile phases, including methanol-water, methanol-water (containing 0.1% formic acid), acetonitrile-water and acetonitrile-water (containing 0.1% formic acid) were examined. Interestingly, sharp peaks were achieved with acetonitrile-water (containing 0.1% formic acid) which proved to be the suitable mobile phase due to the good resolution and mass spectrum response for the most of the analytes. Meanwhile, the effects of column temperature (25, 30, 35, and 40 °C) and flow rate (0.6, 0.8, 1.0 mL/min) were also studied. Finally, column temperature of 30 °C and flow rate of 0.8 mL/min were optimal for qualitative analysis. Likewise, TOF MS parameters, including ion modes, capillary voltage, ion modes and collision energy (CE) were also optimized.

### 2.3. Optimization of UPLC-QqQ MS Conditions

For quantitative, UPLC mobile phases, including water-methanol, water-acetonitrile, methanol-water (containing 0.1% formic acid), and acetonitrile-water (containing 0.1% formic acid) were examined to obtain optimal chromatograms. As a result, good analyte separation was achieved with acetonitrile-water (containing 0.1% formic acid). In addition, the most appropriate precursor ion, daughter ion, cone voltage, collision energy (CE) were adjusted according to each analyte. Finally, the most sensitive transitions in MRM were selected. Glycyrrhetinic acid was chosen as internal standard due to the similar structure, retention time, and ionization response in ESI-MS. The MS data of fourteen related analytes are shown in Table 1.

**Table 1:** Related MS data of fourteen investigated analytes detected on the UPLC-QqQ MS.

Peak Number	Analytes	Quantification Transition (*m*/*z*)	Cone Voltage (V)	Collision Energy (ce)
1	16-oxo-Alisol A-23acetate	529.34→451.34	30	18
2	16-oxo-Alisol A-24acetate	529.34→451.34	30	18
3	Alisol C	487.33→415.35	40	18
4	Alisol F	471.33→339.33	35	12
5	Alisol C-23acetate	529.32→451.32	40	21
6	Alisol L	469.31→397.31	35	22
7	Alisol F 24-acetate	513.31→339.33	35	12
8	Alisol A	473.39→383.39	40	11
9	Alisol A 23-acetate	497.36→365.36	40	16
10	Alisol A 24-acetate	497.36→365.36	40	15
IS	Glycyrrhetinic acid (IS)	471.32→317.28	40	28
11	Alisol G	455.31→437.31	35	10
12	Alisol B	455.31→437.31	40	11
13	Alisol B 23-acetate	497.29→437.29	40	11
14	11-deoxy-Alisol B	479.31→479.31	45	10

### 2.4. Identification of Compounds with HPLC-DAD-Q-TOF MS

**Figure 1:**
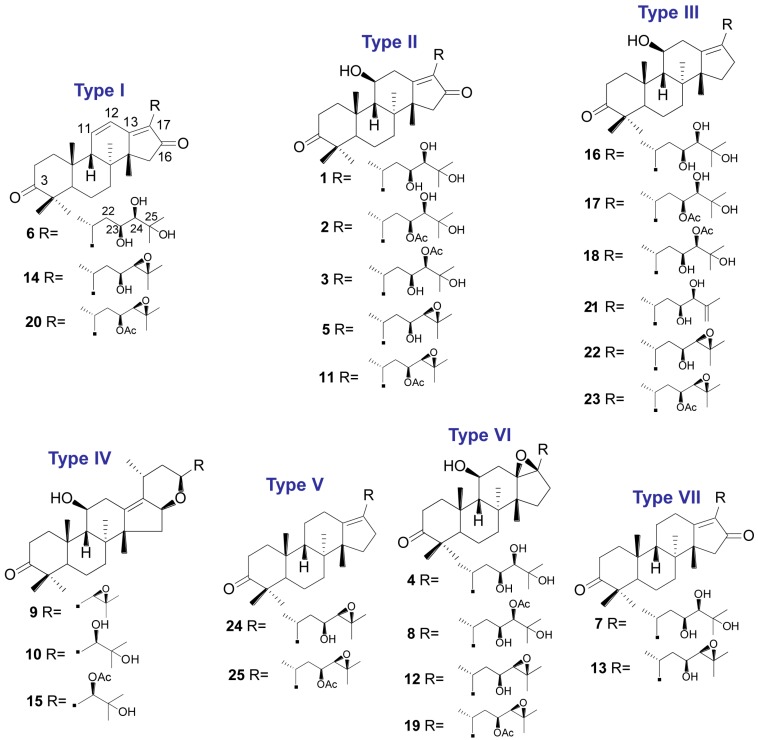
Chemical structures of the identified triterpenoids from AMR.

**Figure 2:**
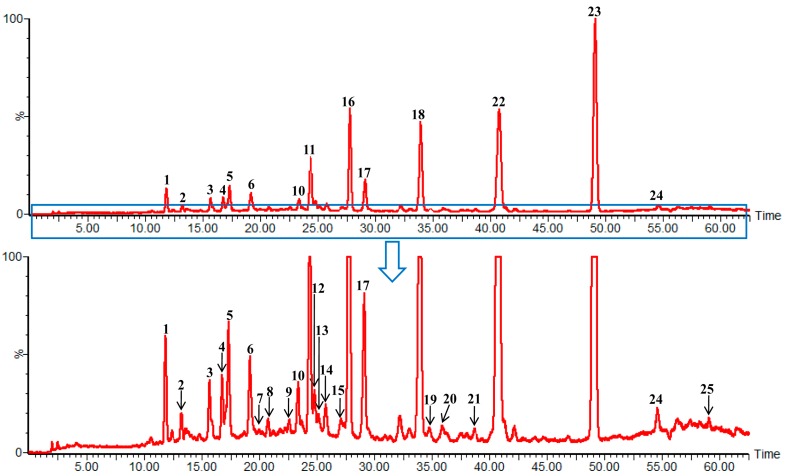
HPLC-DAD-Q-TOF MS total ion chromatogram of AMR in positive ion mode.

**Figure 3:**
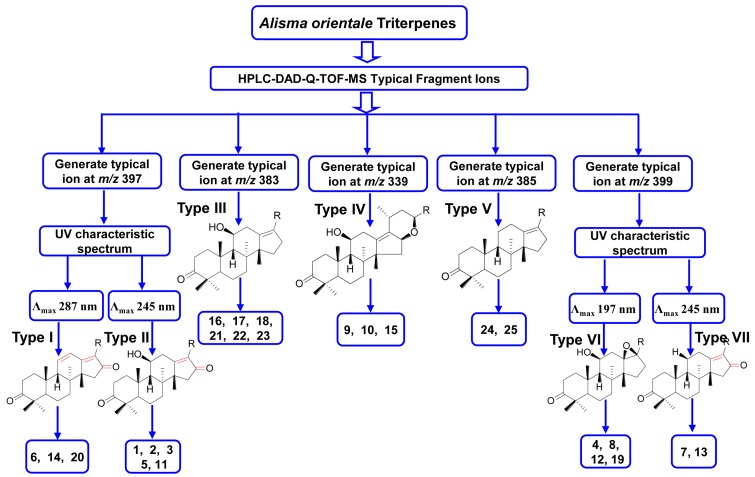
The classification of the 25 compounds based on their structural characteristics.

To qualitatively characterize the chemical constituents of AMR, an HPLC-QTOF MS method was established. As shown in Table 2 and Figure 1, 21 compounds were identified unambiguously by comparing their UV spectra, retention times and accurate masses with data from the corresponding reference standards. For four additional compounds, the structures were tentatively proposed based on UV spectra, accurate masses and fragmentation behaviors. The mass error for molecular ions in all identified compounds was within ± 5 ppm. The total ion chromatogram in the positive mode of AMR is shown in Figure 2. Based on the structural characteristics of the 25 compounds, they were divided into seven types (as shown in Figure 3): type I includes 16-oxo-11-anhydro-alisol A (**6**), alisol L (**14**) and alisol L 23-acetate (**20**); type II includes 16-oxo-alisol A (**1**), 16-oxo-alisol A 23-actetate (**2**), 16-oxo-alisol A 24-actetate (**3**), alisol C (**5**), and alisol C 23-acetate (**11**); type III consists of alisol A (**16**), alisol A 23-actetate (**17**), alisol A 24-actetate (**18**), alisol G (**21**), alisol B (**22**) and alisol B 23-actetate (**23**); type IV comprises 16,23-oxido-alisol B (**9**), alisol F (**10**), and alisol F 24-actetate (**15**); type V includes 11-deoxy-alisol B (**24**) and 11-deoxy-alisol B 23 acetate (**25**); type VI comprises 13,17-epoxy-alisol A (**4**), 13,17-epoxy-alisol A 24-acetate (**8**), 13,17-epoxy-alisol B (**12**) and 13,17-epoxy-alisol B 23-acetate (**19**); type VII includes 11-deoxy-alisol C (**13**) and a new compound named16-oxo-11-deoxy-alisol A (**7**). All 25 triterpenoids exhibited characteristic fragment ion through dissociation rearrangement between the C-23–C-24; in addition, successive or simultaneous losses of H_2_O (18 Da) and/ or HAc (60 Da) were observed clearly in their mass spectra, which yielded product ions of [M + H − H_2_O]^−^, [M + H − HAc]^−^, [M + H − H_2_O − HAc]^−^, [M + H − 2H_2_O − HAc]^−^ (Table 2).

**Table 2:** Characterization of chemical constituents in AMR by HPLC-DAD-Q-TOF MS.

Peak Number	t_R_ (min)	MS^1^	Error (ppm)	Fragment Ions Collected in Positive Mode	Molecular Formula	λ_max_ (nm)	Identity
1	11.81	505.3534 [M + H]^+^	1.0	487.3421 [M + H − H_2_O]^+^, 469.3317 [M + H − 2H_2_O]^+^, 451.3416 [M + H − 3H_2_O]^+^, 415.2845 [M + H − C_4_H_10_O_2_]^+^, 397.2745 [M + H − C_4_H_10_O_2_ − H_2_O]^+^	C_ 30_H_48_O_6_	245	16-oxo-Alisol A ^a^
2	13.19	547.3638 [M + H]^+^	0.5	529.3528 [M + H − H_2_O]^+^, 511.3421 [M + H − 2H_2_O]^+^, 487.3422 [M + H − HAc]^+^, 469.3316 [M + H − Hac − H_2_O]^+^, 415.2848 [M + H − C_6_H_12_O_3_]^+^, 397.2745 [M + H − C_6_H_12_O_3_ − H_2_O]^+^	C_ 32_H_50_O_7_	245	16-oxo-Alisol A 23-actetate ^a^
3	15.63	547.3624 [M + H]^+^	−2.0	529.3529 [M + H − H_2_O]^+^, 511.3429 [M + H − 2H_2_O]^+^, 487.3423 [M + H − HAc]^+^, 469.3318 [M + H – Hac − H_2_O]^+^, 415.2847 [M + H − C_6_H_12_O_3_]^+^, 397.2744 [M + H − C_6_H_12_O_3_ − H_2_O]^+^	C_ 32_H_50_O_7_	245	16-oxo-Alisol A 24-actetate ^a^
4	16.70	507.3687 [M + H]^+^	0.2	489.3577 [M + H − H_2_O]^+^, 471.3471 [M + H − 2H_2_O]^+^, 453.3365 [M + H − 3H_2_O]^+^, 417.3007 [M + H − C_4_H_10_O_2_]^+^, 399.2896 [M + H − C_4_H_10_O_2_ − H_2_O]^+^	C_ 30_H_50_O_6_	-	13,17-epoxy-Alisol A ^a^
5	17.31	487.3423 [M + H]^+^	−0.2	469.3314 [M + H − H_2_O]^+^, 451.3213 [M + H − 2H_2_O]^+^, 415.2650 [M + H − C_4_H_8_O]^+^, 397.2741 [M + H − H_2_O − C_4_H_8_O]^+^, 379.3427 [M + H − 2H_2_O − C_4_H_8_O]^+^	C_ 30_H_46_O_5_	245	Alisol C ^a^
6	19.36	487.3429 [M + H]^+^	1.0	469.3319 [M + H − H_2_O]^+^, 451.3215 [M + H − 2H_2_O]^+^, 397.2742 [M+H-C_4_H_10_O_2_]^+^	C_ 30_H_46_O_5_	287	16-oxo-11-anhydro-Alisol A ^a^
7	19.72	489.3562 [M + H]^+^	−2.7	471.3471 [M + H − H_2_O]^+^, 453.3367 [M + H − 2H_2_O]^+^, 399.2896 [M+H-C_4_H_10_O_2_]^+^	C_ 30_H_48_O_5_	245	16-oxo-11-deoxy-Alisol A
8	21.46	549.3798 [M + H]^+^	1.3	531.3685 [M + H − H_2_O]^+^, 513.3579 [M + H − 2H_2_O]^+^, 489.3585 [M + H − HAc]^+^, 471.3470 [M + H − Hac − H_2_O]^+^, 417.3005 [M + H − C_6_H_12_O_3_]^+^, 399.2896 [M + H − C_6_H_12_O_3_ − H_2_O]^+^	C_ 32_H_52_O_7_	-	13,17-epoxy-Alisol A 24-acetate ^a^
9	23.30	471.3464 [M + H]^+^	−2.1	453.3367 [M + H − H_2_O]^+^, 381.2791 [M + H − C_4_H_8_O − H_2_O]^+^, 339.2684 [M + H − C_4_H_8_O − H_2_O − C_2_H_2_O]^+^	C_ 30_H_46_O_4_	-	16,23-oxido-Alisol B
10	23.37	489.3583 [M + H]^+^	0.6	471.3472 [M + H − H_2_O]^+^, 453.3366 [M + H − 2H_2_O]^+^, 381.2798 [M + H − H_2_O − C_4_H_10_O_2_]^+^, 339.2687 [M + H − H_2_O − C_4_H_10_O_2_ − C_2_H_2_O]^+^	C_ 30_H_48_O_5_	-	Alisol F ^a^
11	24.33	529.3522 [M + H]^+^	−1.3	511.3424 [M + H − H_2_O]^+^, 469.3313 [M + H − HAc]^+^, 451.3212 [M + H − HAc − H_2_O]^+^, 415.2652 [M + H − C_6_H_10_O_2_]^+^,397.2741 [M + H − C_6_H_10_O_2_ − H_2_O]^+^	C_ 32_H_48_O_6_	245	Alisol C 23-acetate ^a^
12	24.75	489.3586 [M + H]^+^	1.2	471.3477 [M + H − H_2_O]^+^, 453.3363 [M + H − 2H_2_O]^+^, 417.3006 [M + H − C_4_H_8_O]^+^, 399.2895 [M + H − C_4_H_8_O − H_2_O]^+^	C_ 30_H_48_O_5_	-	13,17-epoxy-Alisol B ^a^
13	25.46	471.3482 [M + H]^+^	1.7	453.3368 [M + H − H_2_O]^+^, 399.2894 [M + H − C_4_H_8_O]^+^	C_ 30_H_46_O_4_	245	11-deoxy-Alisol C
14	25.91	469.3315 [M + H]^+^	−0.6	451.3216 [M + H − H_2_O]^+^, 397.2745 [M + H − C_4_H_8_O]^+^	C_ 30_H_44_O_4_	287	Alisol L ^a^
15	27.00	531.3694 [M + H]^+^	1.5	513.3585 [M + H − H_2_O]^+^, 495.3598 [M + H − 2H_2_O]^+^, 453.3367 [M + H − H_2_O − HAc]^+^, 435.3592 [M + H − 2H_2_O − HAc]^+^, 381.2791 [M + H − H_2_O − C_6_H_12_O_3_]^+^, 339.2684 [M + H − H_2_O − C_6_H_12_O_3_ − C_2_H_2_O]^+^	C_ 32_H_50_O_6_	-	Alisol F 24-actetate ^a^
16	27.75	491.3731 [M + H]^+^	0.0	473.3630 [M + H − H_2_O]^+^, 455.3524 [M + H − 2H_2_O]^+^, 437.3418 [M + H − 3H_2_O]^+^, 383.2984 [M + H − H_2_O − C_4_H_10_O_2_]^+^	C_ 30_H_50_O_5_	-	Alisol A ^a^
17	29.07	533.3773 [M + H]^+^	0.4	515.3735 [M + H − H_2_O]^+^, 497.3633 [M + H − 2H_2_O]^+^, 455.3526 [M + H − HAc − H_2_O] ^+^, 437.3422 [M + H − HAc − 2H_2_O]^+^, 383.2983 [M + H − C_6_H_12_O_3_ − H_2_O]^+^	C_ 32_H_52_O_6_	-	Alisol A 23-acetate ^a^
18	33.90	533.3848 [M + H]^+^	1.1	515.3736 [M + H − H_2_O]^+^, 497.3634 [M + H − 2H_2_O]^+^, 455.3528 [M + H − HAc − H_2_O] ^+^, 437.3420 [M + H − HAc − 2H_2_O]^+^, 383.2981 [M + H − C_6_H_12_O_3_ − H_2_O]^+^	C_ 32_H_52_O_6_	-	Alisol A 24-acetate ^a^
19	34.95	531.3685 [M + H]^+^	−0.2	513.3581 [M + H − H_2_O]^+^, 495.3476 [M + H − 2H_2_O]^+^, 471.3478 [M + H − HAc]^+^, 453.3367 [M + H − HAc − H_2_O]^+^, 417.3008 [M + H − C_6_H_10_O_2_]^+^, 399.2895 [M + H − C_6_H_10_O_2_ − H_2_O]^+^	C_ 32_H_50_O_6_	-	13,17-epoxy-Alisol B 23-acetate ^a^
20	35.87	511.3418 [M + H]^+^	−1.2	451.3321 [M + H − HAc]^+^, 397.2743 [M + H − C_6_H_10_O_2_]^+^	C_ 32_H_46_O_5_	287	Alisol L 23-acetate ^a^
21	38.87	473.3638 [M + H]^+^	1.5	455.3527 [M + H − H_2_O]^+^, 437.3425 [M + H − 2H_2_O]^+^, 383.2981 [M + H − H_2_O − C_4_H_8_O]^+^	C_ 30_H_48_O_4_	-	Alisol G ^a^
22	40.71	473.3639 [M + H]^+^	1.7	455.3523 [M + H − H_2_O]^+^, 437.2426 [M + H − 2H_2_O]^+^, 383.2982 [M + H − H_2_O − C_4_H_8_O]^+^	C_ 30_H_48_O_4_	-	Alisol B ^a^
23	49.06	515.3739 [M + H]^+^	0.4	497.3631 [M + H − H_2_O]^+^, 479.3519 [M + H − 2H_2_O]^+^, 437.3431 [M + H − H_2_O − HAc]^+^, 383.2983 [M + H − H_2_O − C_6_H_10_O_2_]^+^	C_ 32_H_50_O_5_	-	Alisol B 23-acetate ^a^
24	54.55	457.3675 [M + H]^+^	−1.5	439.3574 [M + H − H_2_O]^+^, 385.3102 [M + H − C_4_H_8_O]^+^	C_ 30_H_48_O_3_	-	11-deoxy-Alisol B ^a^
25	61.12	499.3794 [M + H]^+^	1.4	439.3578 [M + H − HAc]^+^, 385.3105 [M + H − C_6_H_10_O_2_]^+^	C_ 32_H_50_O_4_	-	11-deoxy-Alisol B 23-acetate

t_R_: retention time; MS^1^: qusai-molecular ion; -: maximum UV absorption is below 200 nm; ^a^ Compared with reference compounds.

**Figure 4:**
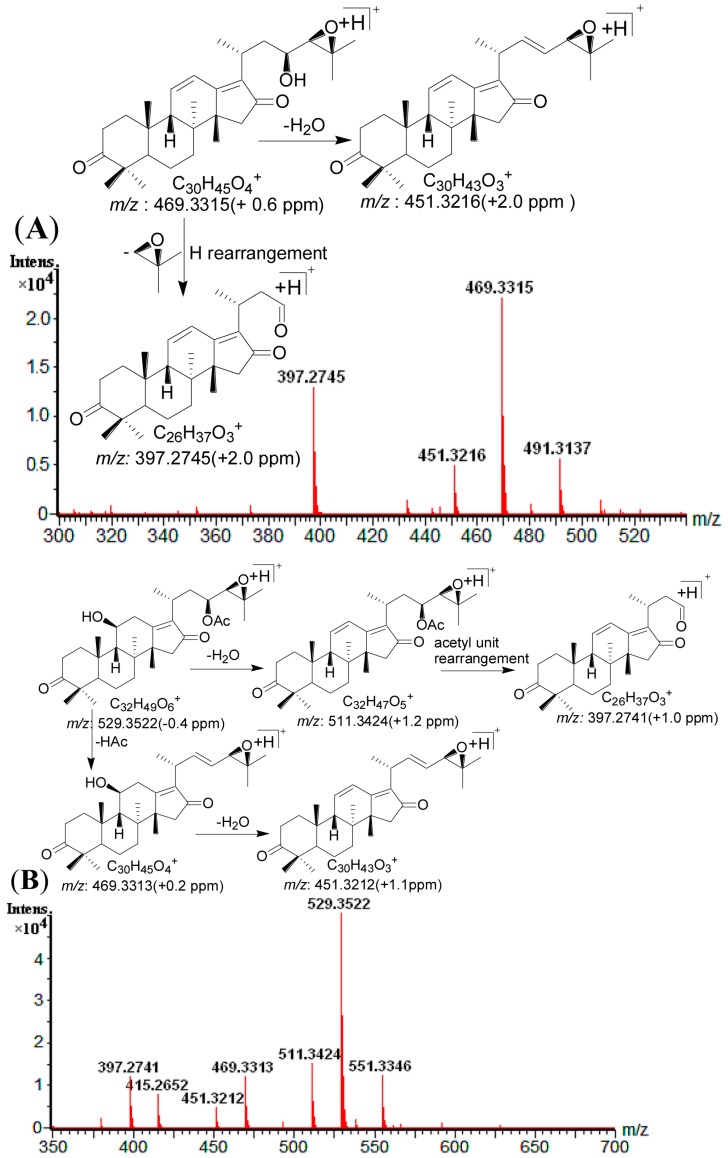

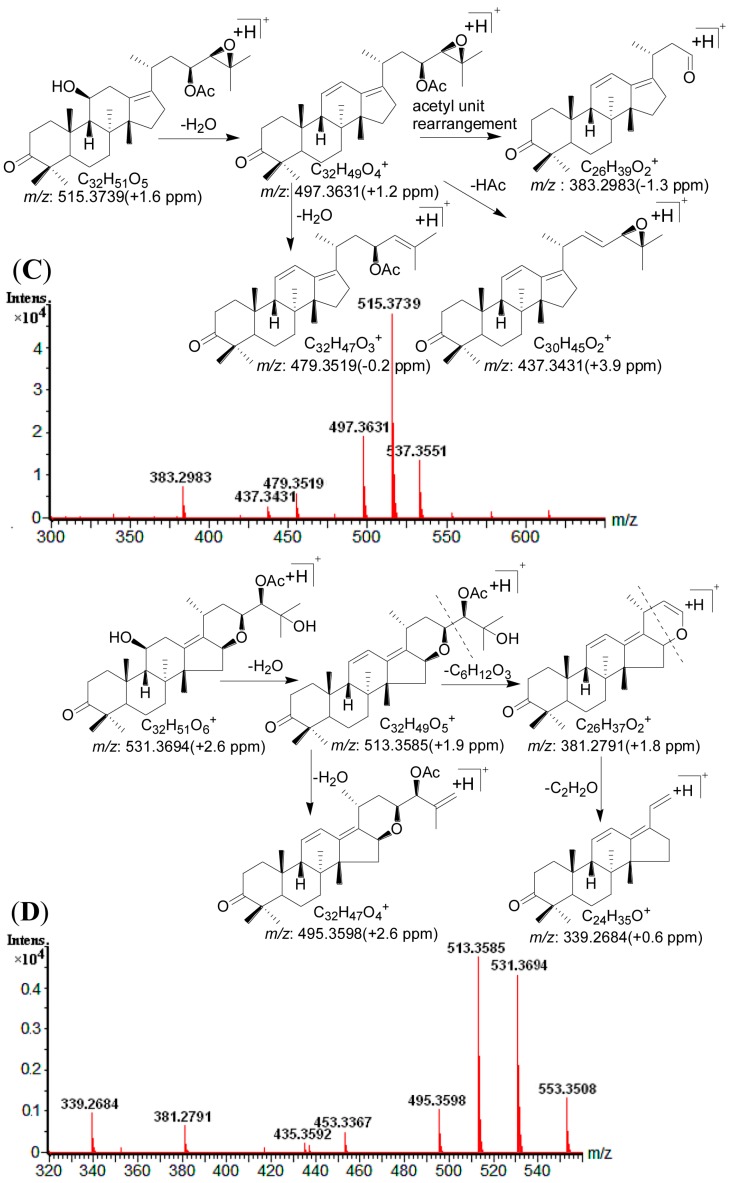

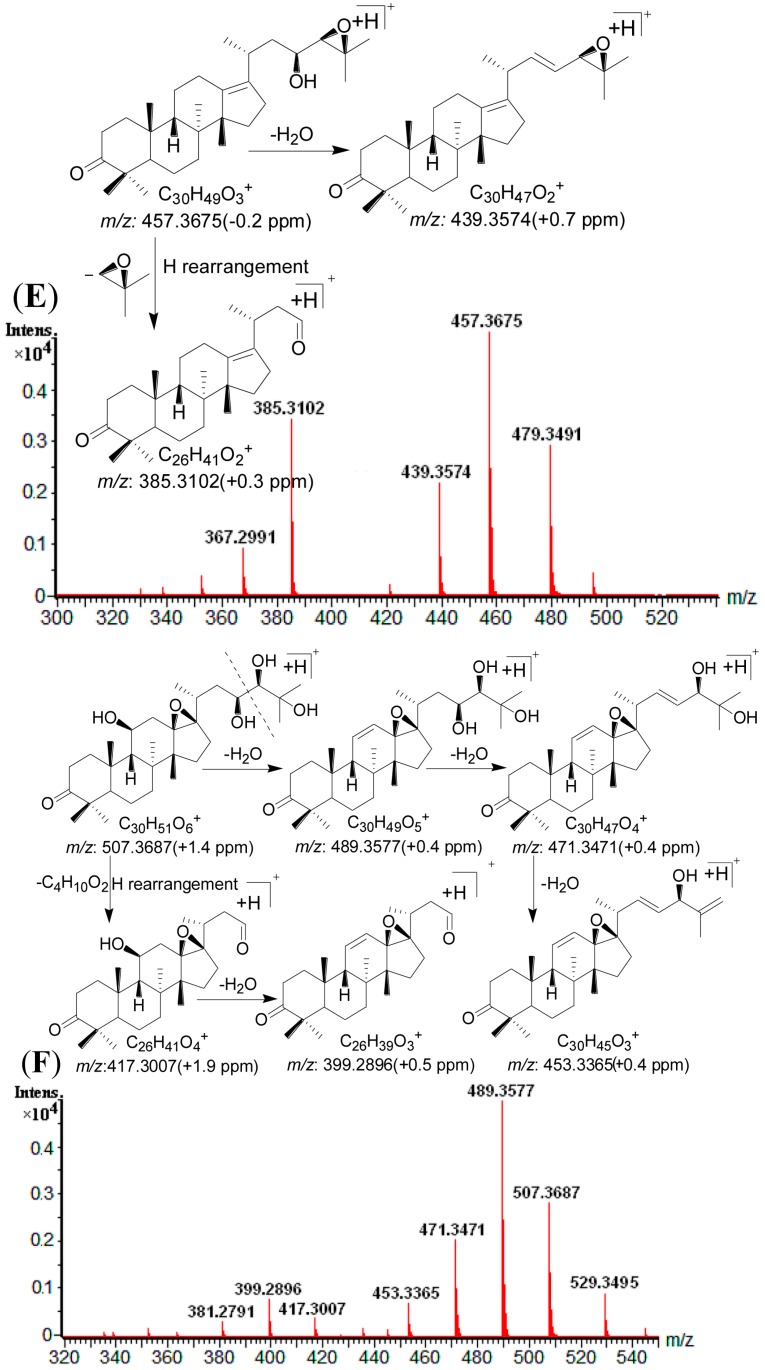

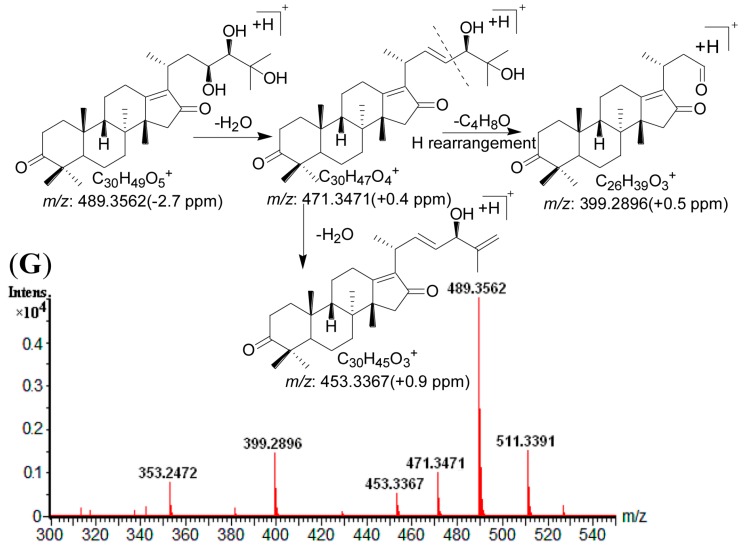
(**A**) The ESI-Q-TOF MS spectra and the proposed fragmentation pathway of alisol L; (**B**) The ESI-Q-TOF MS spectra and the proposed fragmentation pathway of alisol C-23acetate. (C) The ESI-Q-TOF MS spectra and the proposed fragmentation pathway of alisol B-23acetate; (**D**) The ESI-Q-TOF MS spectra and the proposed fragmentation pathway of alisol F-24acetate; (**E**) The ESI-Q-TOF MS spectra and the proposed fragmentation pathway of 11-deoxy-alisol B; (**F**) The ESI-Q-TOF MS spectra and the proposed fragmentation pathway of13,17-epoxy-alisol A; (**G**) The ESI-Q-TOF MS spectra and the proposed fragmentation pathway of 16-oxo-11-deoxy-alisol A.

Type I: Three compounds (**6**, **14** and **20**) were identified as type I triterpenoids by comparison with the corresponding reference standards. They exhibited the same characteristic skeleton ion at *m*/*z* 397.2745 through dissociation rearrangement between the C-23 and C-24, as well as the same maximum UV absorption at 287 nm (λ_max_ = 287 nm) due to the π-π-π conjugated bond at C11, C12, C13, C17 and C16. Alisol L (**14**) (Supplementary Table S1), a representative type I triterpenoid (Figure 4A) displayed [M + Na]^+^ at *m*/*z* 491.3137 and [M + H]^+^ at *m*/*z* 469.3315; it formed the characteristic ion at *m*/*z* 397.2745 and 451.3216 through C-23–C-24 dissociation and 23-OH dehydration, respectively. Owing to the similar fragmentation pathway of Alisol L, compounds **6** and **20** were identified as 16-oxo-11-anhydro-alisol A and alisol L 23-acetate, respectively, based on UV spectra, retention times and MS data, referring to reference standards and previous reports [13].

Type II: All six type II compounds produced the same characteristic skeleton ion at *m*/*z* 397.2745, just liketype I triterpenoids. Compared to type I, they showed no unsaturated bond at C-11-13, which results in maximum UV absorption at245 nm (λ_max_ = 245 nm). Compound **11** (alisol C 23-actetate as an example, Figure 4B) has a protonated molecular ion [M + H]^+^ at *m*/*z* 529.3522 and [M + Na]^+^ at *m*/*z* 551.3346 in accordance with a molecular formula of C_32_H_48_O_6_. The ion at *m*/*z* 511.3424 [M + H − H_2_O]^+^ was generated from [M + H]^+^ by loss of H_2_O (18 Da). The loss of HAc occurred at C-23, forming [M + H − HAc]^+^ at *m*/*z* 469.3313; simultaneous loss of H_2_O (18 Da) yielded a product ion at *m*/*z* 451.3212 [M + H − HAc − H_2_O]^+^; the C-23–C-24 dissociation produced [M + H − C_6_H_10_O_2_]^+^ at *m*/*z* 415.2652, and subsequent loss of H_2_O (18 Da) yielded a product ionat *m*/*z* 397.2741 [M + H − H_2_O − C_6_H_10_O_2_]^+^. Other type II compounds **1**, **2**, **3** and **5** showed similar fragmentation behaviors to Alisol C 23-actetate, and were identified as 16-oxo-alisol A, 16-oxo-alisol A 23-actetate, 16-oxo-alisol A 24-actetate, and alisol C, respectively. All were further confirmed by comparison with reference standards.

Type III: Type III triterpenoids usually generate a typical ion at *m*/*z* 383.2983. The characteristic compound **23** (alisol B 23-acetate, Figure 4C) showed [M + Na]^+^ at *m*/*z* 537.3551 and [M + H]^+^ at *m*/*z* 515.3739. An [M + H − H_2_O]^+^ ion at *m*/*z* 497.3631, [M + H − 2H_2_O]^+^ at *m*/*z* 479.3519, and [M + H − H_2_O − HAc]^+^ at *m*/*z* 437.3431 were detected in the mass spectrum through several dehydrations or deacetylations. The typical ion [M + H − H_2_O − C_6_H_10_O_2_]^+^ at *m*/*z* 383.2983 was generated by dissociation of C-23–C-24 and loss of H_2_O at C-11 (18 Da). Analogously, compounds **16**, **17**, **18**, **21**, and **22** were identified as alisol A, alisol A 23-actetate, alisol A 24-actetate, alisol G and alisol B, based on MS data of reference standards.

Type IV: Type IV consists of compounds **9**, **10** and **15**, which produced the typical characteristic ion at *m*/*z* 339.2684. Compound **15** (take alisol F 24-actetate, as an example in Figure 4D) exhibited [M + H]^+^ at *m*/*z* 531.3694 and several dehydration or deacetylation ions at *m*/*z* 513.3585 [M + H − H_2_O]^+^, 495.3598 [M + H − 2H_2_O]^+^, and 435.3592 [M + H − 2H_2_O − HAc]^+^. Ion *m*/*z* 381.2791 was formed by C11-dehydration and the C-23–C-24 dissociation generated [M + H − H_2_O − C_6_H_12_O_3_]^+^, with further loss of C_2_H_2_O generating an ion at *m*/*z* 339.2684 [M + H − H_2_O − C_6_H_12_O_3_ − C_2_H_2_O]^+^. Compound **10** was similar to alisol F 24-actetate except for deacetylation, and was further confirmed by alisol F standard data. Similarly, compound **9** was tentatively identified as 16,23-oxido-alisol B by fragmentation behavior as previously reported [13].

Type V: Compounds **24** and **25** were identified as type V triterpenoids by their similar fragmentation behaviors. Compound **24** was identified as 11-deoxy-alisol B by comparing with its standard, which has a 24,25-oxide ring in the structure. It showed [M + Na]^+^ at *m*/*z* 479.3491 and [M + H]^+^ at *m*/*z* 457.3675. The ions at *m*/*z* 439.3574 and *m*/*z* 385.3102 were obtained by C-23-OH dehydration and hydrogen rearrangement, respectively (as shown in Figure 4E). Analogously, compound **25** was tentatively identified as 11-deoxy-alisol B 23 acetate [13].

Type VI: Type VI compounds showed a unique 13,17-oxide ring and produced a typical ion at *m*/*z* 399.2896. Their maximum UV absorption was below 200 nm due to the absence of conjugated bonds. For example (Figure 4F), 13,17-epoxy-alisol A (**4**) generated an [M + H]^+^ ion at *m*/*z* 507.3687, and several dehydration ions *m*/*z* 489.3577 [M + H − H_2_O]^+^, 471.3471 [M + H − 2H_2_O]^+^ and 453.3365 [M + H − 3H_2_O]^+^. The C-23–C-24 dissociation and C11-dehydration yielded the typical ion at *m*/*z* 399.2899 [M + H − C_4_H_10_O_2_ − H_2_O]^+^. Compounds **8**, **12**, and **19** which showed similar fragmentation behaviors to 13,17-epoxy-alisol A, were identified as 13,17-epoxy-alisol A 24-acetate, 13,17-epoxy-alisol B and 13,17-epoxy-alisol B 23-acetate, respectively. All were further confirmed by comparison with their reference standards.

Type VII: Compounds **7** and **13** produced a typical ion at *m*/*z* 399.2896, just like type VI triterpenoids. Compared to type VI compounds, they showed an unsaturated bond at C-11–C-13, which resulted in maximum UV absorption at 245 nm (λ_max_ = 245 nm). Compound **7** (an example, Figure 4G), showed an [M + H]^+^ ion at *m*/*z* 489.3562 in accordance with a molecular formula of C_30_H_48_O_5_. Two dehydrations produced ions at *m*/*z* 471.3471 [M + H − H_2_O]^+^ and 453.3367 [M + H − 2H_2_O]^+^. [M + H − HAc]^+^ was not detected, suggesting a non-acetyl triterpenoid. The dissociation of the C-23–C-24 bond by hydrogen rearrangement at C-23-OH yielded the product ion at *m*/*z* 399.2896 [M + H − C_4_H_10_O_2_]^+^. The tentatively identified compound **7** was named 16-oxo-11-deoxy-alisol A. In order to further confirm its structure, it was purified by preparative high performance liquid chromatography coupled with mass spectrometry detector autopurification system (p-HPLC-MS). Finally, 2.6 mg power of compound **7** (t_R_ 15.4 min) was prepared from the acetonitrile extract of AMR by p-HPLC-MS (Xbridge C_18_ 19 mm ´ 150 mm, 5 μm column Waters, Manchester, UK; acetonitrile (A)–H_2_O (B), gradient elution: 52%–52% A at 0–5 min, 55%–65% A at 5–20 min, 65%–90% A at 20–25 min, 52%–52% A at 26–40 min, 12 mL/min; detected ion set at 489). And its structure was further confirmed by ^1^H-NMR and ^13^C-NMR spectrometry data [22] (Supplementary Table S2, Figure S2–S5).

Compound **13** exhibited an [M + H]^+^ ion at *m*/*z* 471.3482. The ion at *m*/*z* 453.3368 [M + H − H_2_O]^+^ was generated by eliminating one H_2_O (18 Da) with the hydroxyl groups. The dissociation of the C-23–C-24 bond via hydrogen rearrangement at C-23-OH yielded the product ion at *m*/*z* 399.2894 [M + H − C_4_H_8_O]^+^. Compound **13** was tentatively identified as 11-deoxy-alisol C by fragmentation behavior comparing with previous reports [13,23].

### 2.5. Method Validation

The linear calibration curves were produced by plotting the ratios of the peak areas of each standard to IS against the concentration of each analyte. Acceptable linear correlation in these conditions was confirmed by correlation coefficients (*r*, 0.998 0–0.999 9). The LODs (S/N = 3) and LOQs (S/N = 10) for the 14 standard analytes were 1.01–9.23 and 3.91–27.4 ng/mL, respectively, indicating that this method is sensitive for the quantitative determination of major components in AMR samples (Table 3).

**Table 3:** Retention time, linear regression data, LODs and LOQs of fourteen analytes for UPLC-QqQ MS quantification.

Analytes	t_R_ (min)	Calibration Curve	Linear Range μg/mL	*r* (*n* = 6)	LOD ng/mL	LOQ ng/mL
16-oxo-Alisol A 23-acetate	1.48	*Y* = 15.87*X* − 0.014	0.008–0.103	0.9999	1.17	3.91
16-oxo-Alisol A 24-acetate	1.71	*Y* = 17.21*X* − 0.002	0.009–0.138	0.9990	1.82	6.05
Alisol C	1.95	*Y* = 5.005*X* − 0.043	0.037–1.490	0.9980	3.63	12.1
Alisol F	2.78	*Y* = 2.496*X* − 0.013	0.062–1.125	0.9989	2.45	8.17
Alisol C 23-acetate	2.87	*Y* = 9.118*X* − 0.029	0.045–1.352	0.9990	1.70	5.67
Alisol L	3.10	*Y* = 12.22*X* − 0.054	0.024–0.652	0.9996	1.68	5.59
Alisol F 24-acetate	3.25	*Y* = 2.160*X* − 0.038	0.078–1.060	0.9990	7.01	21.0
Alisol A	3.38	*Y* = 3.125*X* − 0.016	0.066–3.333	0.9982	1.61	5.35
Alisol A 23-acetate	3.54	*Y* = 3.441*X* − 0.017	0.042–1.080	0.9993	1.73	5.75
Alisol A 24-acetate	4.05	*Y* = 3.505*X* − 0.010	0.049–3.240	0.9990	1.01	4.37
Alisol G	4.61	*Y* = 6.788*X* − 0.019	0.026–0.525	0.9980	3.63	12.1
Alisol B	4.84	*Y* = 1.635*X* − 0.013	0.197–5.444	0.9982	3.40	10.6
Alisol B 23-acetate	5.67	*Y* = 2.678*X* − 0.023	0.294–9.790	0.9993	3.04	10.1
11-deoxy-Alisol B	6.36	*Y* = 2.584*X* − 0.058	0.089–1.740	0.9990	9.23	27.4

The RSD values of intra-day and inter-day variations, repeatability and stability of the target components were 1.18%–3.79%, 1.53%–3.96%, 2.21%–4.25%, and 1.32%–3.97% respectively. The recovery rate of the fourteen standards varied from 98.11% to 103.8% (RSD ≤ 4.06%). These results are summarized in Table 4. In conclusion, the developed method had good linearity, precision, repeatability, stability, and accuracy.

**Table 4:** Precision, repeatability, stability, and recovery of fourteen analytes (*n* = 6).

Analytes	Precision for Standard Solution (*n* = 6)		Repeatability from Real Samples		Stability (%)	Accuracy	
	Inter-Day RSD (%)	Intra-Day RSD (%)	RSD (%, *n* = 6)	Mean (mg/g)		Recovery (%)	RSD (%)
16-oxo-Alisol A 23-acetate	3.14	2.73	3.17	0.011	97.54 ± 2.49	100.2	3.10
16-oxo-Alisol A 24-acetate	3.52	3.55	3.17	0.006	99.23 ± 2.39	101.7	2.02
Alisol C	3.94	3.79	3.97	0.128	98.57 ± 3.72	99.62	2.44
Alisol F	1.53	1.46	2.21	0.182	101.23 ± 2.73	103.8	2.53
Alisol C 23-acetate	2.14	3.60	3.02	0.174	96.22 ± 3.82	98.11	2.24
Alisol L	3.96	2.29	2.47	0.012	98.93 ± 1.59	101.3	4.06
Alisol F 24-acetate	2.22	1.28	2.86	0.089	99.75 ± 1.98	100.8	2.21
Alisol A	2.15	1.82	3.49	0.595	100.24 ± 3.52	98.17	2.15
Alisol A 23-acetate	2.14	1.81	4.03	0.105	96.88 ± 3.59	103.8	3.14
Alisol A 24-acetate	2.85	2.23	3.96	0.110	97.59 ± 3.61	102.0	3.84
Alisol G	2.08	3.22	3.01	0.031	97.92 ± 2.02	99.87	2.58
Alisol B	3.70	3.00	4.25	1.086	98.36 ± 1.30	99.34	3.20
Alisol B 23-acetate	2.22	1.48	2.31	1.465	100.56 ± 1.61	101.0	2.82
11-deoxy-Alisol B	2.04	1.18	2.22	0.104	99.37 ± 3.19	101.2	3.04

### 2.6. Sample Analysis

**Figure 5:**
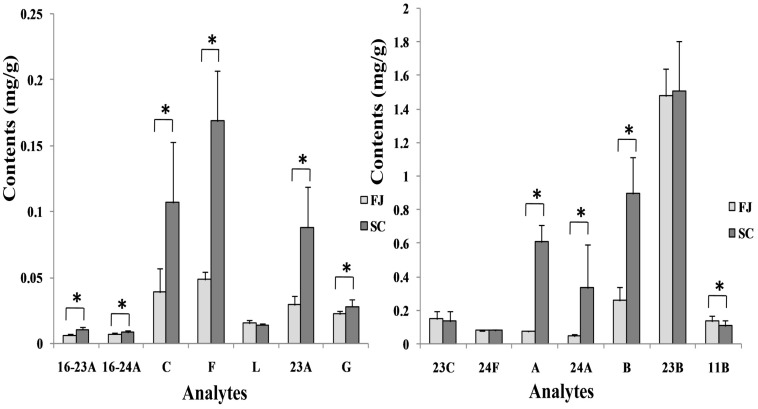
Contents (mg/g) of the fourteen triterpenoids in AMR. 16-23A: 16-oxo-alisol A 23-acetate, 16-24A: 16-oxo-alisol A 24-acetate, C: alisol C, F: alisol F, 23C: alisol C 23-acetate, L: alisol L, 24F: alisol F 24-acetate, A: alisol A, 23A: alisol A 23-acetate, 24A: alisol A 24-acetate, G: alisol G, B: alisol B, 23B: alisol B 23-acetate, 11B: 11-deoxy-alisol B. FJ: Fujian Samples, SC: Sichuan Samples, Student’s *t*-test was used for the statistical analysis. * *p* < 0.05.

This developed analytical method was successfully applied to simultaneously determine the fourteen major components in forty-three AMR batches obtained from two major GAP bases. The UPLC-QqQ MS MRM chromatograms in the positive ion mode of the 14 components are shown in Figure 5. Their contents are summarized in Table 5. The results indicated different contents for these triterpenes in crude drugs from different origins. Among these compounds, alisol B and alisol B 23-acetate were dominant compounds in all samples, at amounts of 0.104–1.232 mg/g and 1.131–2.032 mg/g, respectively. Comparing the crude drugs, obvious differences could be observed between Fujian and Sichuan samples: 16-oxo-alisol A 23-acetate, 16-oxo-alisol A 24-acetate, alisol C, alisol F, alisol A, alisol A 23-acetate, alisol A 24-acetate, alisol G and alisol B were found at higher amounts in Sichuan samples but 11-doxy-alisol B was lower compared with Fujian samples. And the other compounds, including alisol C 23-acetate, alisol L, alisol F 24-acetate and alisol B 23-acetate showed no significant difference between the two sources (Figure 5).

**Table 5:** Contents (mg/g) of the fourteen triterpenoids in AMR (*n* = 3).

NO.	Sample	1	2	3	4	5	6	7	8	9	10	11	12	13	14
S1	Fujian 1	0.009	0.008	0.055	0.041	0.121	0.012	0.082	0.067	0.029	0.052	0.020	0.307	1.420	0.180
S2	Fujian 2	0.006	0.007	0.068	0.044	0.113	0.013	0.081	0.069	0.032	0.061	0.020	0.325	1.546	0.177
S3	Fujian 3	0.006	0.007	0.027	0.063	0.118	0.013	ND	0.075	0.030	0.069	0.024	0.118	1.560	0.183
S4	Fujian 4	0.006	0.008	0.025	0.047	0.219	0.018	0.08	0.070	0.021	0.028	0.020	0.324	1.216	0.124
S5	Fujian 5	0.005	0.005	0.023	0.046	0.204	0.018	0.081	0.081	0.023	0.028	0.021	0.291	1.780	0.122
S6	Fujian 6	0.006	0.007	0.027	0.049	0.244	0.020	0.085	0.075	0.024	0.030	0.025	0.342	1.502	0.131
S7	Fujian 7	0.007	0.008	0.070	0.057	0.141	0.014	ND	0.079	0.031	0.061	0.023	0.246	1.459	0.127
S8	Fujian 8	0.006	0.009	0.045	0.048	0.153	0.013	0.081	0.080	0.032	0.062	0.021	0.237	1.451	0.095
S9	Fujian 9	0.006	0.006	0.022	0.042	0.113	0.014	0.082	0.069	0.047	0.053	0.027	0.104	1.273	0.133
S10	Fujian 10	0.005	0.007	0.024	0.048	0.099	0.018	0.083	0.082	0.024	0.029	0.022	0.333	1.769	0.121
S11	Fujian 11	0.008	0.008	0.054	0.042	0.121	0.013	0.082	0.068	0.029	0.051	0.020	0.308	1.420	0.181
S12	Fujian 12	0.007	0.007	0.067	0.044	0.114	0.013	0.081	0.069	0.032	0.061	0.020	0.326	1.546	0.178
S13	Fujian 13	0.006	0.007	0.027	0.063	0.118	0.013	ND	0.075	0.030	0.069	0.024	0.118	1.560	0.183
S14	Fujian 14	0.005	0.008	0.026	0.048	0.219	0.019	0.08	0.070	0.021	0.029	0.021	0.324	1.216	0.124
S15	Fujian 15	0.006	0.005	0.024	0.046	0.205	0.018	0.082	0.081	0.024	0.028	0.021	0.291	1.780	0.122
S16	Fujian 16	0.006	0.007	0.027	0.049	0.244	0.020	0.085	0.076	0.024	0.030	0.025	0.343	1.300	0.132
S17	Fujian 17	0.007	0.008	0.070	0.057	0.141	0.014	ND	0.079	0.031	0.061	0.024	0.246	1.459	0.127
S18	Fujian 18	0.007	0.009	0.045	0.048	0.153	0.013	0.081	0.080	0.032	0.062	0.021	0.238	1.452	0.095
S19	Fujian 19	0.006	0.006	0.022	0.043	0.114	0.014	0.083	0.069	0.047	0.054	0.027	0.104	1.273	0.133
S20	Fujian 20	0.005	0.007	0.024	0.048	0.099	0.018	0.083	0.082	0.024	0.029	0.022	0.333	1.569	0.122
S21	Fujian 21	0.007	0.008	0.044	0.049	0.153	0.013	0.082	0.080	0.032	0.062	0.022	0.237	1.451	0.095
S22	Sichuan 1	0.011	0.008	0.207	0.184	0.202	0.013	0.085	0.802	0.115	0.543	0.038	0.905	1.418	0.163
S23	Sichuan 2	0.010	0.007	0.145	0.184	0.138	0.013	0.079	0.594	0.111	0.803	0.035	1.208	2.002	0.138
S24	Sichuan 3	0.009	0.007	0.137	0.213	0.131	0.013	0.079	0.568	0.105	0.807	0.032	1.232	2.032	0.166
S25	Sichuan 4	0.011	0.006	0.128	0.182	0.174	0.012	0.089	0.595	0.105	0.110	0.031	1.086	1.465	0.104
S26	Sichuan 5	0.008	0.006	0.108	0.131	0.065	0.017	ND	0.594	0.035	0.102	0.022	0.784	1.438	0.109
S27	Sichuan 6	0.010	0.008	0.077	0.156	0.069	0.013	0.082	0.567	0.102	0.279	0.025	0.740	1.163	0.104
S28	Sichuan 7	0.012	0.009	0.034	0.211	0.143	0.012	ND	0.756	0.107	0.271	0.026	1.016	1.217	0.061
S29	Sichuan 8	0.015	0.011	0.069	0.221	0.241	0.013	0.081	0.707	0.114	0.297	0.029	0.525	1.513	0.123
S30	Sichuan 9	0.012	0.007	0.104	0.118	0.114	0.016	ND	0.445	0.047	0.104	0.024	0.663	1.821	0.052
S31	Sichuan 10	0.012	0.010	0.073	0.109	0.182	0.015	0.090	0.491	0.062	0.155	0.021	0.912	1.131	0.072
S32	Sichuan 11	0.010	0.009	0.206	0.184	0.202	0.014	0.085	0.802	0.114	0.544	0.038	0.906	1.419	0.164
S33	Sichuan 12	0.011	0.010	0.145	0.185	0.138	0.013	0.079	0.594	0.111	0.803	0.035	1.208	2.001	0.139
S34	Sichuan 13	0.009	0.008	0.138	0.213	0.131	0.014	0.081	0.568	0.105	0.807	0.032	1.231	2.032	0.166
S35	Sichuan 14	0.011	0.010	0.128	0.183	0.174	0.012	0.089	0.596	0.105	0.111	0.031	1.086	1.465	0.104
S36	Sichuan 15	0.009	0.007	0.108	0.131	0.065	0.017	ND	0.594	0.035	0.102	0.023	0.784	1.439	0.109
S37	Sichuan 16	0.010	0.008	0.078	0.156	0.069	0.014	0.082	0.567	0.103	0.279	0.025	0.741	1.663	0.105
S38	Sichuan 17	0.011	0.009	0.034	0.212	0.144	0.012	ND	0.757	0.107	0.271	0.026	1.016	1.217	0.061
S39	Sichuan 18	0.015	0.011	0.069	0.221	0.241	0.013	0.081	0.707	0.114	0.297	0.029	0.525	1.513	0.123
S40	Sichuan 19	0.012	0.007	0.105	0.118	0.114	0.016	ND	0.446	0.048	0.105	0.024	0.663	1.522	0.053
S41	Sichuan 20	0.013	0.011	0.073	0.109	0.183	0.015	0.091	0.491	0.062	0.155	0.021	0.913	1.131	0.072
S42	Sichuan 21	0.008	0.007	0.109	0.132	0.065	0.017	ND	0.595	0.036	0.103	0.023	0.784	1.438	0.109
S43	Sichuan 22	0.010	0.008	0.077	0.156	0.069	0.014	0.082	0.567	0.102	0.279	0.025	0.741	1.164	0.105

1 16-oxo-alisol A 23-acetate; 2 16-oxo-alisol A 24-acetate; 3 alisol C; 4 alisol F; 5 alisol C 23-acetate; 6 alisol L; 7 alisol F 24-acetate; 8 alisol A; 9 alisol A 23-acetate; 10 alisol A 24-acetate; 11 alisol G; 12 alisol B; 13 alisol B 23-acetate; 14 11-deoxy-alisol B; ND: Below the LOD.

## 3. Experimental Section

### 3.1. Materials and Standards, Reagents

Forty-three batches of AMR samples were collected from two major good agricultural practice (GAP) bases approved by State Food and Drug Administration of China (SFDA). These crude drugs were identified by Prof. Shui-Sheng Wu. Voucher specimens were deposited in the College of Pharmacy, Fujian University of Traditional Chinese Medicine.

Acetonitrile, methanol, and formic acid (HPLC grade) for LC analysis were purchased from Merck (Darmstadt, Germany). Deionized water was prepared using a system from Millipore (Bedford, MA, USA); other solvents were of analytical grade. Twenty-one standards were previously isolated and purified from AMR in our laboratory; their structures were confirmed by MS, ^1^H-NMR spectrometry, and ^13^C-NMR spectrometry [24,25]. Fourteen of them, including 16-oxo-alisol A 23-acetate (purity, 98.5%), 16-oxo-alisol A 24-acetate (purity, 99.0%), alisol C (purity, 98.5%), alisol F (purity, 99.6%), alisol C 23-acetate (purity, 99.5%), alisol L (purity, 99.0%), alisol F 24-acetate (purity, 99.2%), alisol A (purity, 99.3%), alisol A 23-acetate (purity, 98.7%), alisol A 24-acetate (purity, 99.2%), alisol G (purity, 99.0%), alisol B (purity, 99.7%), alisol B 23-acetate (purity, 99.8%) and 11-deoxy-alisol B (purity, 99.0%), were used for quantitation; their purity were determined by HPLC-DAD-ELSD. 16-oxo-alisol A (purity, 98.0%), alisol L 23-acetate, 13,17-epoxy-alisol A (purity, 99.0%), 16-oxo-11-anhydro-alisol A (purity, 99.1%), 13,17-epoxy-alisol A 24-acetate (purity, 98.5%), 13,17-epoxy-alisol B (purity, 99.0%) and 13,17-epoxy-alisol B 23-acetate (purity, 99.2%) were used for qualitative analysis due to their low amounts; Glycyrrhetinic acid (purity, 98.5%, internal standard, IS) was purchased from National Institute for the Control of Pharmaceutical and Biological Products (Beijing, China).

### 3.2. Preparation of Standard Solution and Samples

All standards were individually dissolved in acetonitrile to approx. 1 mg/mL. The stock solution for each quantitative analyte was further diluted with acetonitrile to achieve a series of working solutions used to establish the calibration curves. All solutions were stored at 4 °C, and filtered through a 0.22 μm membrane before use.

Forty-three batches of AMR samples were ground to fine powder and well mixed. Exactly 0.20 g powder was weighted and ultrasonicated for 30 min in 25 mL acetonitrile. The extraction solution was centrifuged at 16,000× *g* for 10 min, and the supernatant filtered through a 0.22 μm membrane for analysis.

The internal standard glycyrrhetinic acid was prepared in acetonitrile to a final concentration of about 0.5 μg/mL. 500 μL of this working solution were added to 500 μL of each sample solution or mixed standard solution, vortexed and filtered through a 0.22 μm membrane before analysis.

### 3.3. Qualitative Chromatographic Conditions

Qualitative analysis of AMR was performed on a Shimadzu HPLC system (Kyoto, Japan) coupled with a Bruker micrOTOF-Q II mass spectrometer (Bremen, Germany). A LC-20A pump, CTO-20A column thermostat, SIL-20A auto injector, and SPD-M20A DAD detector were included in the HPLC system. Separation was achieved on an Ultimate XB-C_18_ column (4.6 ´ 150 mm, 5 μm, Welch) maintained at 30 °C. The mobile phase consisted of water (containing 0.1% formic acid, phase A) and acetonitrile (phase B) with the following gradient program: 35% B at 0–5 min, 35%–55% B at 5–20 min, 55%–65% B at 20–35 min, 65%–75% B at 35–45 min, 75%–90% B at 45–55 min 90% B at 55–60 min, 90%–35% B at 60–61 min, 35% B at 61–75 min. The flow rate was kept at 0.8 mL/min, with an injection volume of 10 μL.

Mass spectra were acquired in the positive ion mode with electrospray ionization (ESI) source. The ESI-MS condition was optimized: capillary voltage, 3.5 kV; nebulizer pressure, 2.0 bar; dry gas (N_2_) flow rate, 4 L/min; dry gas temperature, 180 °C; spectrum rate, 1.7 Hz; scan range, *m*/*z* 50–1000; funnel 1 and 2, 200.0 Vpp; hexapole Rf, 120.0 Vpp; quadrupole ion energy, 3.0 eV; collision Rf, 350.0 Vpp. Argon was used as the collision gas, with collision energy set at 10–50 eV to obtain ion fragment data. External instrument calibration was applied daily before sample analysis in order to achieve an acceptable accuracy threshold at 5 ppm. Accurate mass data of the molecular ions were processed by the Data Analysis software (Bruker Daltonics, Bremen, Germany).The preparative high-performance liquid chromatography coupled with a mass spectrometer detector auto-purification system (Waters, Manchester, UK), including a Waters 2545 apparatus equipped with a 2767 fraction collector, a Waters SQD2 quadrupole mass spectrometer, and a Waters Xbridge C_18_ (19 mm ´ 150 mm, 5 μm, Waters) column, was used for preparation.

### 3.4. Quantitative Chromatographic Conditions

Quantitative chromatographic analysis was performed on a Waters TQ QqQ MS system (Waters, Manchester, UK) equipped with an online vacuum degasser, an autosampler, a binary pump and a thermostatted column compartment. Chromatographic separation was carried out on a Waters CORTECS UPLC C_18_ (2.1 mm ´ 100 mm, 1.6 μm, Waters) at 45 °C. The mobile phase consisted of water (A) and acetonitrile (B), with the following elution gradient program: 45%–85% B at 0–5.0 min, 85%–95% B at 5.0–6.0 min, 95%–95% B at 6.0–6.5 min, 45%–45% B at 6.5–8.5 min. The flow rate was kept at 0.30 mL/min, with an injection volume of 2 μL.

**Figure 6:**
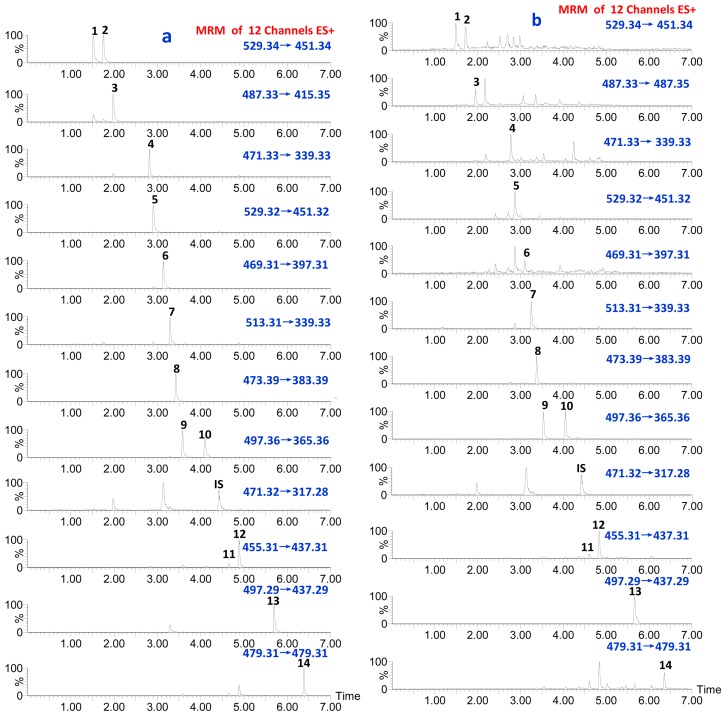
UPLC-QqQ MS MRM chromatogram in positive ion mode of (**a**) fourteen target standards and (**b**) sample of AMR.

Mass spectrometry was performed on tandem mass spectrometer with an electrospray ionization (ESI) source. Nitrogen was used as nebulizer, curtain and heater gas; Argon was used as collision gas. The nebulizergas was set at 500 L/h at 200 °C in the positive ion MRM mode. The cone gas was used at a flow rate of 50 L/h, with the source temperature set at 150 °C. The capillary voltage was 3.0 kV. Most proper cone voltageand collision energy (CE) were selected according to each analyte. UPLC-QqQ MS MRM chromatogram in positive ion mode of (a) fourteen target standards and (b) sample of AMR in Figure 6.

### 3.5. Validation of the Quantitative Method

Seven concentrations of calibration standards were made to establish calibration curves by plotting the ratios of the peak areas of each standard to IS against the analyte concentration. A series of diluted standard solutions were prepared to determine the limits of detection (LODs) and limits of quantification (LOQs), corresponding to signal-to-noise ratios (S/N) of about 3 and 10, respectively, for each analyte under the described UPLC-QqQ MS conditions.

Intra-day and inter-day precisions were determined to evaluate the precision of the developed method. For intra-day precision, the mixed standard solutions were analyzed in six replicates within one day, while for inter-day precision, the mixed standard solutions were examined in duplicates for three consecutive days. Both variations were expressed as relative standard deviation (RSD). Six samples were prepared independently to assess repeatability. In order to evaluate sample stability, each sample solution was measured at seven time points (0, 4, 8, 12, 16, 20, and 24 h) within 24 h. The recovery was used to investigate the accuracy of the method, determined by spiking mixed standard solutions to known amounts of AMR samples. Then the mixture was extracted and analyzed. The recovery rates were calculated according to the following equation:

recovery rates = (detected amount − original amount) 100%/added amount
(1)

## 4. Conclusions

In this study, The chemical profile of AMR was thoroughly and systematically investigated by HPLC-QTOF-MS. Twenty-five compounds were unambiguously or tentatively identified Based on the results of qualitative analysis, a stable and reliable quantitative method using UPLC-QqQ was established, which has been successfully applied to simultaneously determine 14 compounds in 43 batches of AMR. This qualitative identification and quantitative analysis method provides an essential data for further chemical or pharmacological studies of AMR.

## Supplementary Materials

Supplementary materials can be accessed at: http://www.mdpi.com/1420-3049/20/08/13958/s1.

## References

[1] Dan H., Wu J., Peng M., Hu X., Song C., Zhou Z., Yu S., Fang N. (2011). Hypolipidemic effects of alismatis rhizome on lipid profile in mice fed high-fat diet. Saudi Med. J..

[2] Li Q., Qu H. (2012). Study on the hypoglycemic activities and metabolism of alcohol extract of *Alismatis Rhizoma*. Fitoterapia.

[3] Lee J.H., Kwon O.S., Jin H.G., Woo E.R., Kim Y.S., Kim H.P. (2012). The rhizomes of *Alisma orientale* and alisol derivatives inhibit allergic response and experimental atopic dermatitis. Biol. Pharm. Bul..

[4] Lee S., Kho Y., Min B., Kim J., Na M., Kang S., Maeng H., Bae K. (2001). Cytotoxic triterpenoides from *Alismatis Rhizoma*. Arch. Pharm. Res..

[5] Feng Y.L., Chen H., Tian T., Chen D.Q., Zhao Y.Y., Lin R.C. (2014). Diuretic and anti-diuretic activities of the ethanol and aqueous extracts of *Alismatis Rhizoma*. J. Ethnopharmacol..

[6] Xu W., Li T., Qiu J.F., Wu S.S., Huang M.Q., Lin L.G. (2015). Anti-Proliferative Activities of Terpenoids Isolated from *Alisma orientalis* and their Structure-Activity Relationships. Anti-Cancer Agents Med. Chem..

[7] Jin H.G., Jin Q., Ryun Kim A., Choi H., Lee J.H., Kim Y.S., Lee D.G., Woo E.R. (2012). A new triterpenoid from *Alisma orientale* and their antibacterial effect. Arch. Pharm. Res..

[8] Adams M., Gschwind S., Zimmermann S., Kaiser M., Hamburger M. (2011). Renaissance remedies: Antiplasmodial protostane triterpenoids from *Alisma plantago-aquatica* L. (Alismataceae). J. Ethnopharmacol..

[9] Han C.W., Kang E.S., Ham S.A., Woo H.J., Lee J.H., Seo H.G. (2012). Antioxidative effects of *Alisma orientale* extract in palmitate-induced cellular injury. Pharm. Biol..

[10] Han C.W., Kwun M.J., Kim K.H., Choi J.Y., Oh S.R., Ahn K.S., Lee J.H., Joo M. (2013). Ethanol extract of *Alismatis Rhizoma* reduces acute lung inflammation by suppressing nf-kappab and activating nrf2. J. Ethnopharmacol..

[11] Yoshikawa M., Yamaguchi S., Chatani N., Nishino Y., Matsuoka T., Yamahara J., Murakami N., Matsuda H., Kubo M. (1994). Crude drugs from aquatic plants. III Quantitative analysis of triterpene constituents in *Alismatis Rhizoma* by means of high performance liquid chromatography on the chemical change of the constituents during *Alismatis Rhizoma* processing. J. Pharm. Soc. Jpn..

[12] Luo Z., Zhou A., Zhang C., Zhang M. (2010). Simultaneous determination of four alisols in *Rhizoma Alismatis* by RP-HPLC. China J. Chin. Mater. Med..

[13] Liu X., Li S.L., Zhou Y., Song J.Z., Zheng Y.F., Peng G.P., Xu H.X. (2010). Characterization of protostane triterpenoids in *Alisma orientalis* by ultra-performance liquid chromatography coupled with quadrupole time-of-flight mass spectrometry. Rapid Commun. Mass Spectrom..

[14] Shaw L.H., Chen W.M., Tsai T.H. (2013). Identification of multiple ingredients for a traditional Chinese medicine preparation (bu-yang-huan-wu-tang) by liquid chromatography coupled with tandem mass spectrometry. Molecules.

[15] Julianti T., Oufir M., Hamburger M. (2014). Quantification of the antiplasmodial alkaloid carpaine in papaya (*Carica papaya*) leaves. Planta Med..

[16] Huang M., Zhao H., Xu W., Chu K., Hong Z., Peng J., Chen L. (2013). Rapid simultaneous determination of twelve major components in pien tze huang by ultra-performance liquid chromatography coupled with triple quadrupole mass spectrometry. J. Sep. Sci..

[17] Kanakis P., Termentzi A., Michel T., Gikas E., Halabalaki M., Skaltsounis A.L. (2013). From olive drupes to olive oil. An HPLC-orbitrap-based qualitative and quantitative exploration of olive key metabolites. Planta Med..

[18] Song Y.X., Liu S.P., Jin Z., Qin J.F., Jiang Z.Y. (2013). Qualitative and quantitative analysis of andrographis paniculata by rapid resolution liquid chromatography/time-of-flight mass spectrometry. Molecules.

[19] Ding J.Y., Liu X.X., Xiong D.M., Ye L.M., Chao R.B. (2014). Simultaneous determination of thirteen aminoalcohol-diterpenoid alkaloids in the lateral roots of aconitum carmichaeli by solid-phase extraction-liquid chromatography-tandem mass spectrometry. Planta Med..

[20] Wang X., Sun H., Zhang A., Wang P., Han Y. (2011). Ultra-performance liquid chromatography coupled to mass spectrometry as a sensitive and powerful technology for metabolomic studies. J. Sep. Sci..

[21] Dong J., Zhu Y., Gao X., Chang Y., Wang M., Zhang P. (2013). Qualitative and quantitative analysis of the major constituents in Chinese medicinal preparation dan-lou tablet by ultra high performance liquid chromatography/diode-array detector/quadrupole time-of-flight tandem mass spectrometry. J. Pharm. Biomed. Anal..

[22] Nakajima Y., Satoh Y., Ida Y., Shoji J. (1994). Terpenoids of *Alisma orientale* rhizome and the crude drug *Alismatis Rhizoma*. Phytochemistry.

[23] Fukuyama Y., Pei-Wu G., Rei W., Yamada T., Nakagawa K. (1988). 11-deoxyalisol c and alisol d: New protostane-type triterpenoids from *Alisma plantago-aquatica*. Planta Med..

[24] Makabel B., Zhao Y., Wang B., Bai Y., Zhang Q., Wu L., Lv Y. (2008). Stability and structure studies on alisol a 24-acetate. Chem. Pharm. Bull..

[25] Yoshikawa M., Yamaguchi S., Matsuda H., Tanaka N., Yamahara J., Murakami N. (1994). Crude drugs from aquatic plants. V. On the constituents of *Alismatis Rhizoma*. (3). Stereostructures of water-soluble bioactive sesquiterpenes, sulfoorientalols a, b, c, and d, from Chinese *Alismatis Rhizoma*. Chem. Pharm. Bull..

