# Supplementary material for: Qualitative and Quantitative Analysis of Major Triterpenoids in Alismatis Rhizoma by High Performance Liquid Chromatography/Diode-Array Detector/Quadrupole-Time-of-Flight Mass Spectrometry and Ultra-Performance Liquid Chromatography/Triple Quadrupole Mass Spectrometry
Source: Molecules. 2015 Jul 31;20(8):13958–81. doi: 10.3390/molecules200813958 (PMC6331801; doi:10.3390/molecules200813958)
Supplement: Supplementary file 1 [file molecules-20-13958-s001.pdf]

Supplementary Material

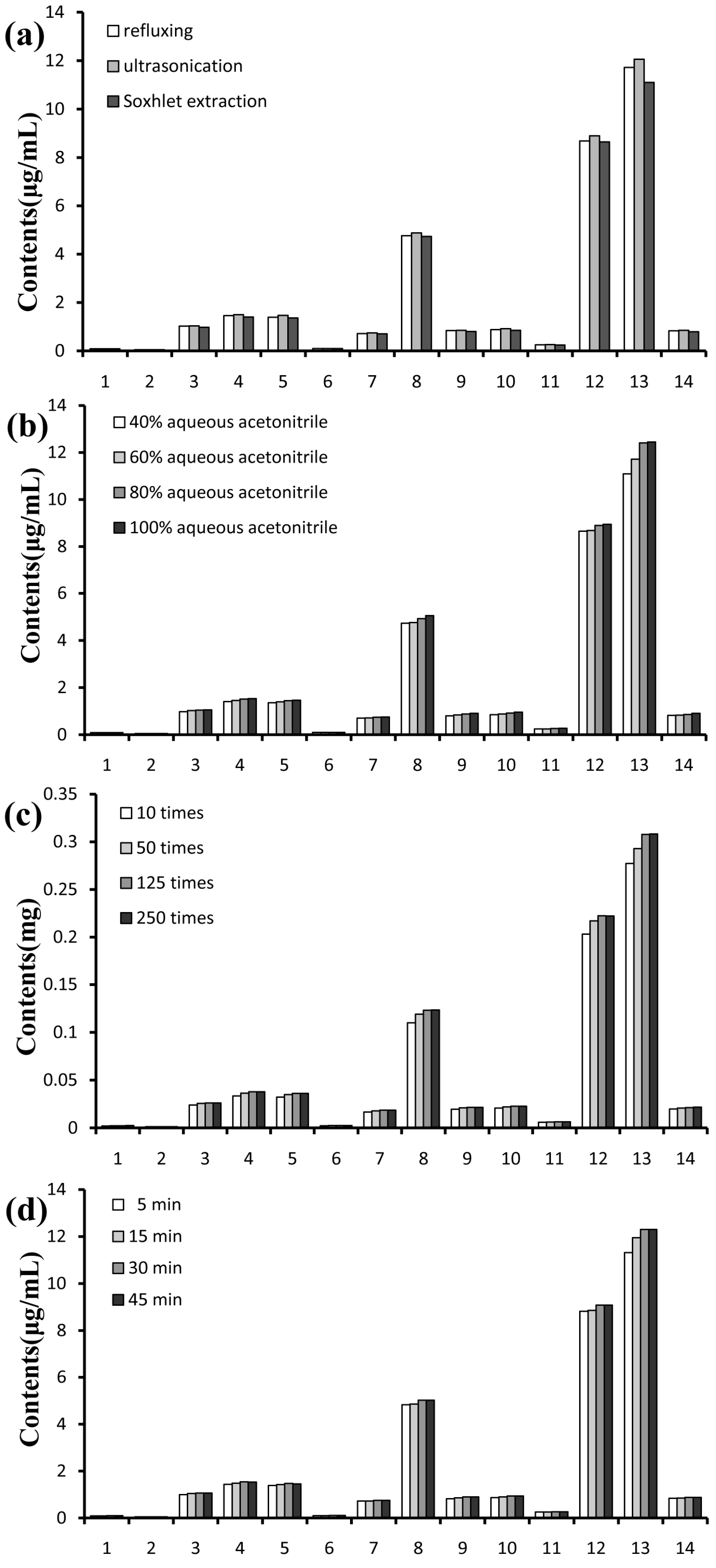

Figure S1. Extraction efficiencies of the typical compounds **1**–**14** by different extract methods (**a**); different extraction solvents (**b**); different times the volume of 100% acetonitrile (**c**); and different extraction time (**d**).

Table S1. Typical DAD spectra from 200 nm to 400 nm for 25 compounds.

NO.	t_R (min)	Molecular Formula	Characteristic Skeleton	DAD Spectra	λ_{max} (nm)
6	19.36	$C_{30}H_{46}O_5$	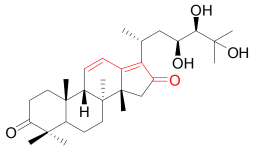	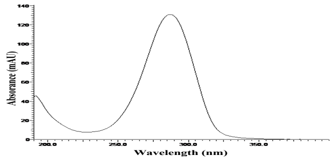	287
14	25.91	$C_{30}H_{44}O_4$	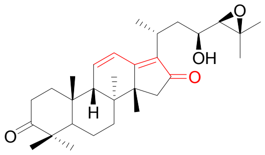	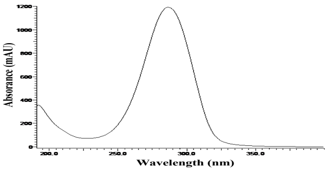	287
20	35.87	$C_{32}H_{46}O_5$	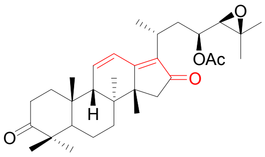	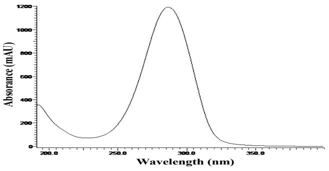	287
1	11.81	$C_{30}H_{48}O_6$	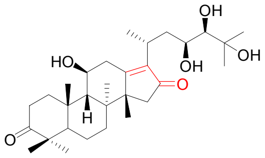	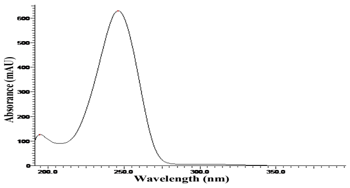	245
2	13.19	$C_{32}H_{50}O_7$	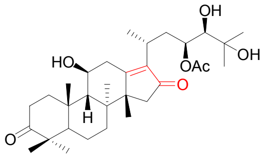	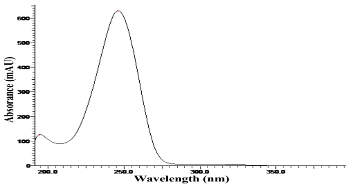	245
3	15.63	$C_{32}H_{50}O_7$	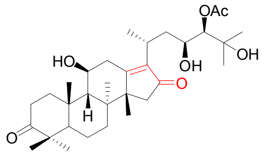	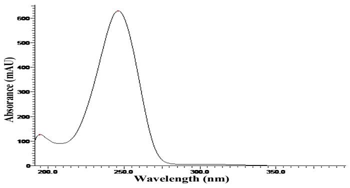	245
5	17.31	$C_{30}H_{46}O_5$	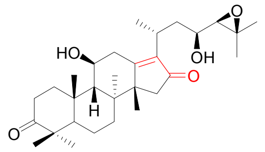	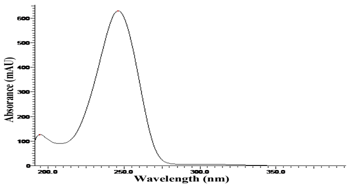	245
11	24.33	$C_{32}H_{48}O_6$	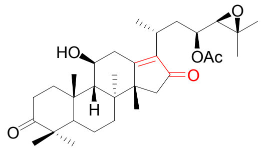	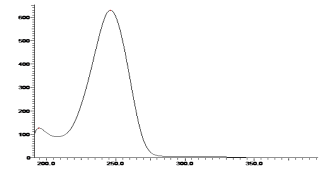	245
16	27.75	$C_{30}H_{50}O_5$	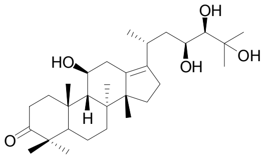	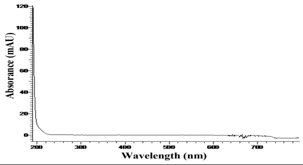	-

Table S1. *Cont.*

NO.	t_R (min)	Molecular Formula	Characteristic Skeleton	DAD Spectra	λ_{max} (nm)
17	29.07	$C_{32}H_{52}O_6$	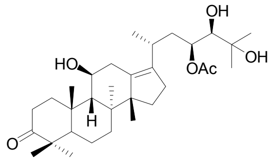	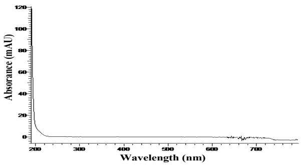	-
18	33.90	$C_{32}H_{52}O_6$	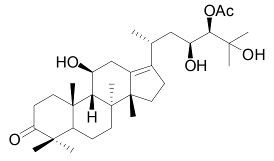	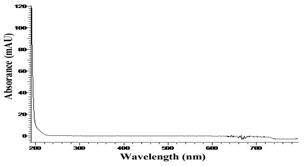	-
21	38.87	$C_{30}H_{48}O_4$	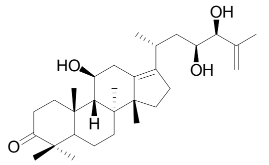	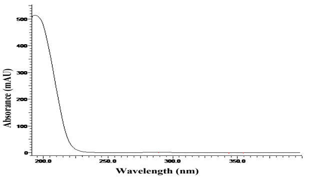	-
22	40.71	$C_{30}H_{48}O_4$	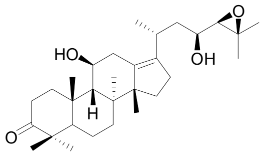	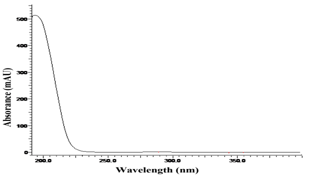	-
23	49.06	$C_{32}H_{50}O_5$	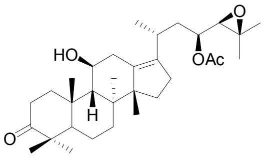	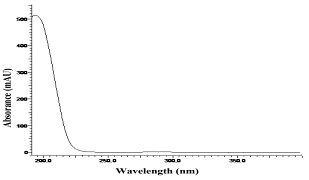	-
9	23.30	$C_{30}H_{46}O_4$	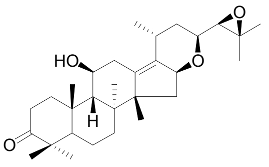	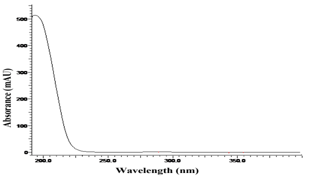	-
10	23.37	$C_{30}H_{48}O_5$	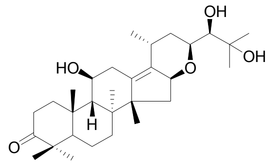	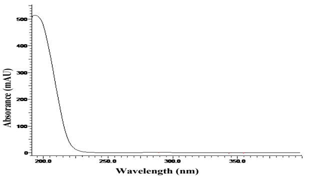	-
15	27.00	$C_{32}H_{50}O_6$	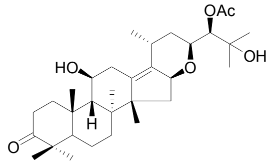	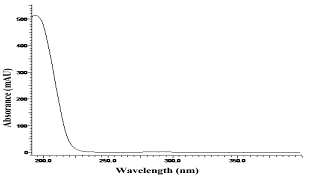	-
24	54.55	$C_{30}H_{48}O_3$	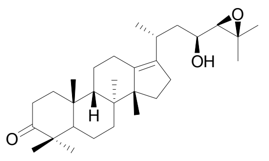	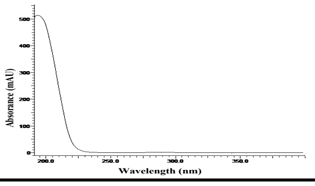	-

Table S1. *Cont.*

NO.	t_R (min)	Molecular Formula	Characteristic Skeleton	DAD Spectra	λ_{max} (nm)
25	61.12	$C_{32}H_{50}O_4$	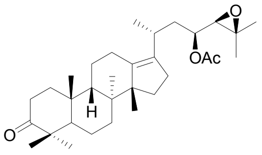	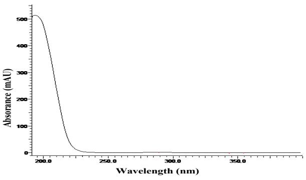	-
4	16.70	$C_{30}H_{50}O_6$	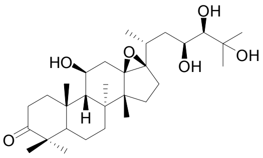	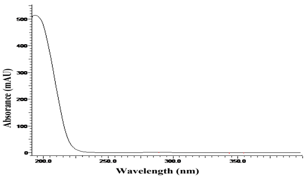	-
8	21.46	$C_{32}H_{52}O_7$	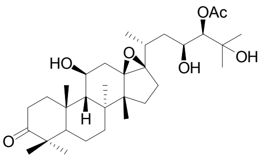	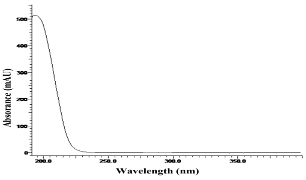	-
12	24.75	$C_{30}H_{48}O_5$	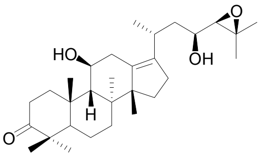	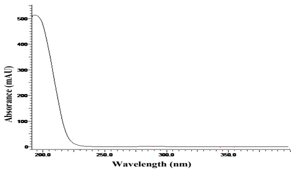	-
19	34.95	$C_{32}H_{50}O_6$	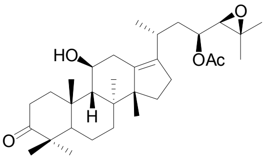	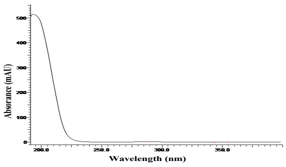	-
7	19.72	$C_{30}H_{48}O_5$	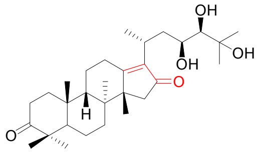	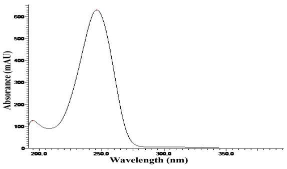	245
13	25.46	$C_{30}H_{46}O_4$	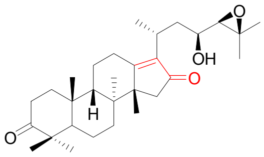	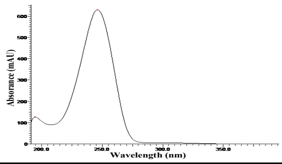	245

-: maximum UV absorption is below 200 nm.

Table S2. ^{1}H- and ^{13}C-NMR data for compound **7** (16-oxo-11-deoxy-alisol A) in $CDCl_3$ (δ in ppm, *J* in Hz).

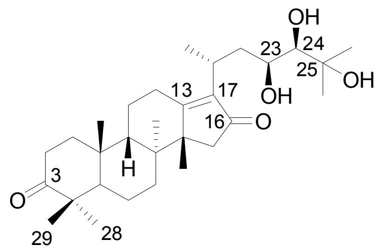

Position	11-deoxy-alisol A [1]		16-oxo-11-deoxy-alisol A		
	δ_H	δ_C	δ_H	δ_C	DEPT
1		31.2		31.74 t	CH_2
2		34.3		34.53 t	CH_2
3		219.2		219.31 s	C
4		47.1		46.98 s	C
5		48.4		48.05 d	CH
6		20.4		19.97 t	CH_2
7		34.0		33.58 t	CH_2
8		40.7		40.51 s	C
9		44.2		42.71 d	CH
10		36.5		36.22 s	C
11		22.8		22.17 t	CH_2
12		23.10		24.99 t	CH_2
13		139.5		180.40 s	C
14		57.50		50.44 s	C
15		32.00		45.43 t	CH_2
16		29.10		209.96 s	C
17		134.2		140.23 s	C
18		23.3		23.35 q	CH_3
19		23.6		23.67 q	CH_3
20		28.7		26.98 d	CH
21		20.0	1.23(d,6.56)	19.51 q	CH_3
22		40.3		40.29 t	CH_2
23	3.80(dd,3.3,9.0)	69.7	3.63(d,10.5)	69.37 d	CH
24	3.03(br s)	77.6	3.02(br s)	77.36 d	CH
25		74.0		73.69 s	C
26		26.4		25.47 q	CH_3
27		27.5		26.34 q	CH_3
28		29.5		29.25 q	CH_3
29		20.5		19.69 q	CH_3
30		23.8		22.02 q	CH_3

[1] Nakajima Y, Satoh Y, Ida Y *et al.* Terpenoids of Alisma orientale rhizome and the crude drug alismatis rhizoma. *Phytochemistry* **1994**, *36*, 119–127.

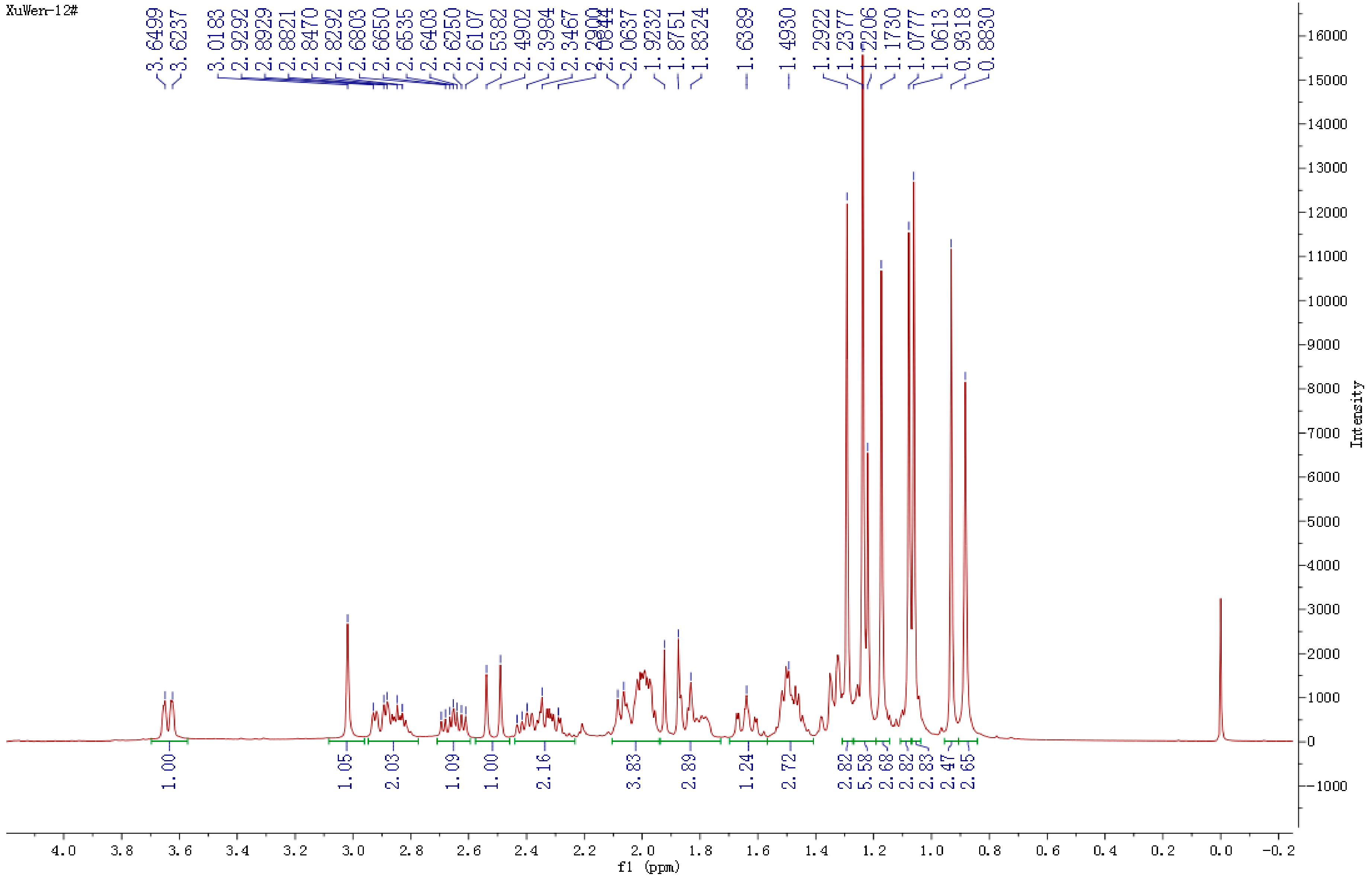

Figure S2. ^{1}H-NMR of 16-oxo-11-deoxy-alisol A.

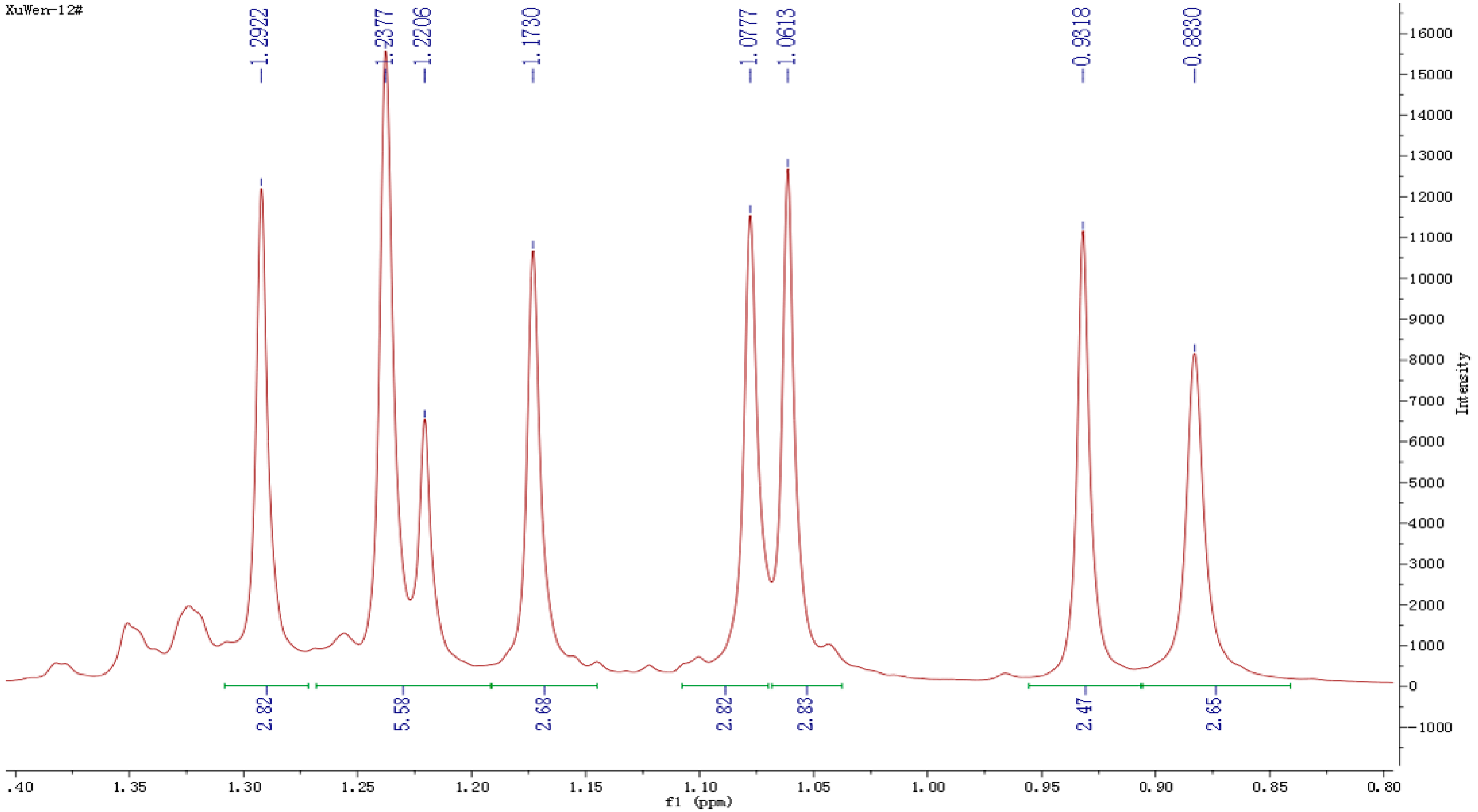

Figure S3. ^{1}H-NMR of 16-oxo-11-deoxy-alisol A—Expansion.

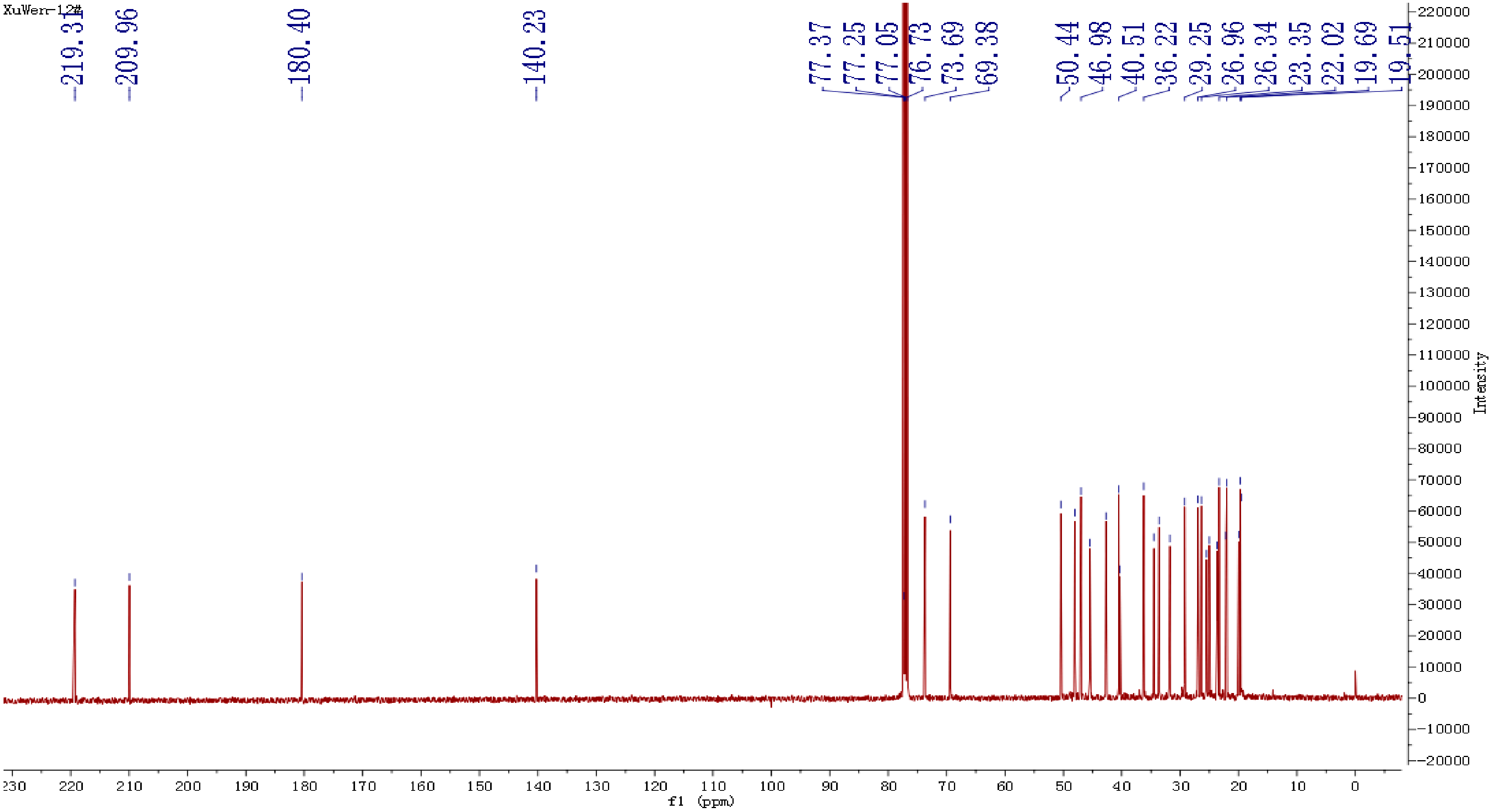

Figure S4. ^{13}C-NMR of 16-oxo-11-deoxy-alisol A.

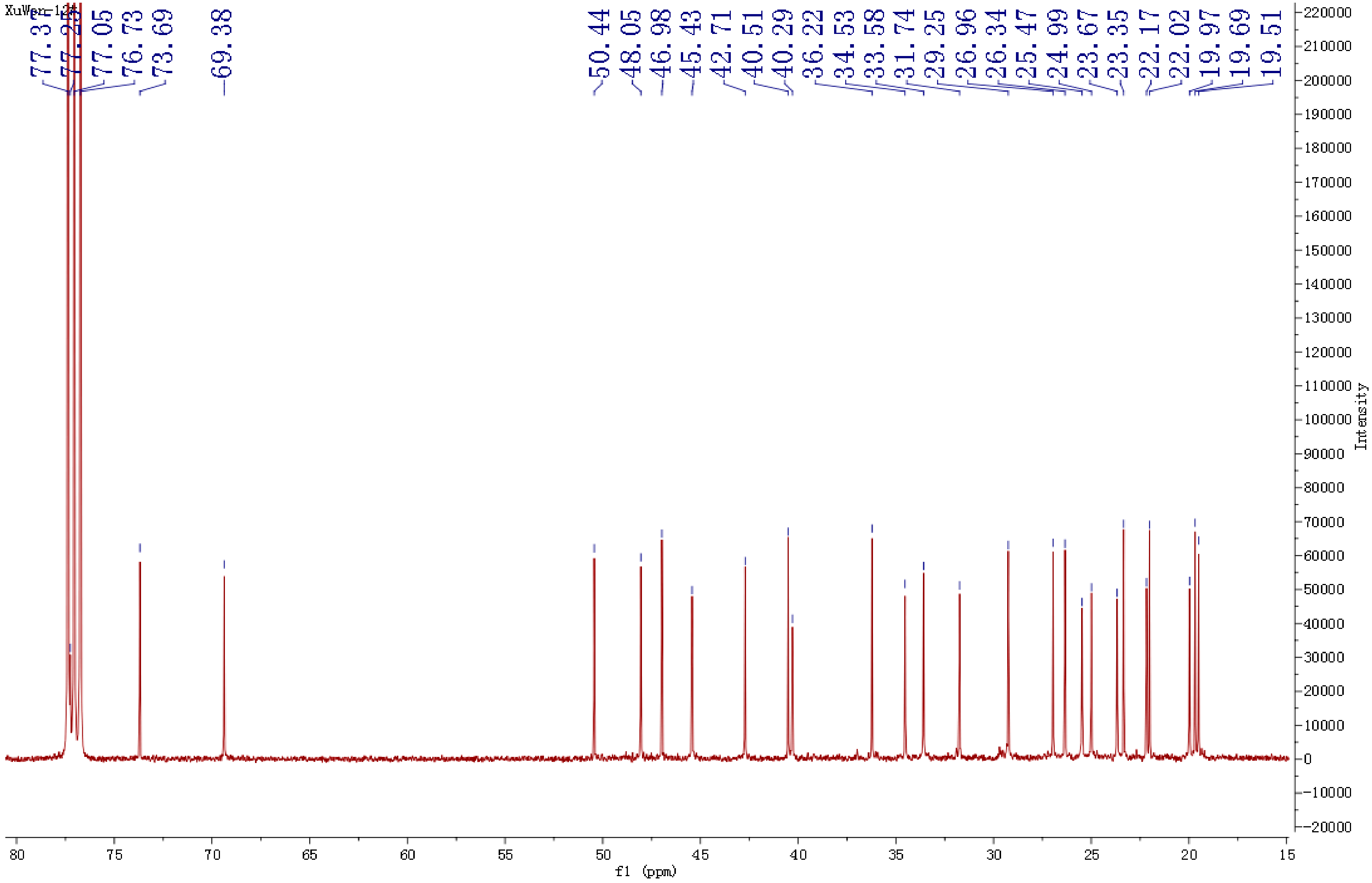

Figure S5. ^{13}C-NMR of 16-oxo-11-deoxy-alisol A—Expansion.

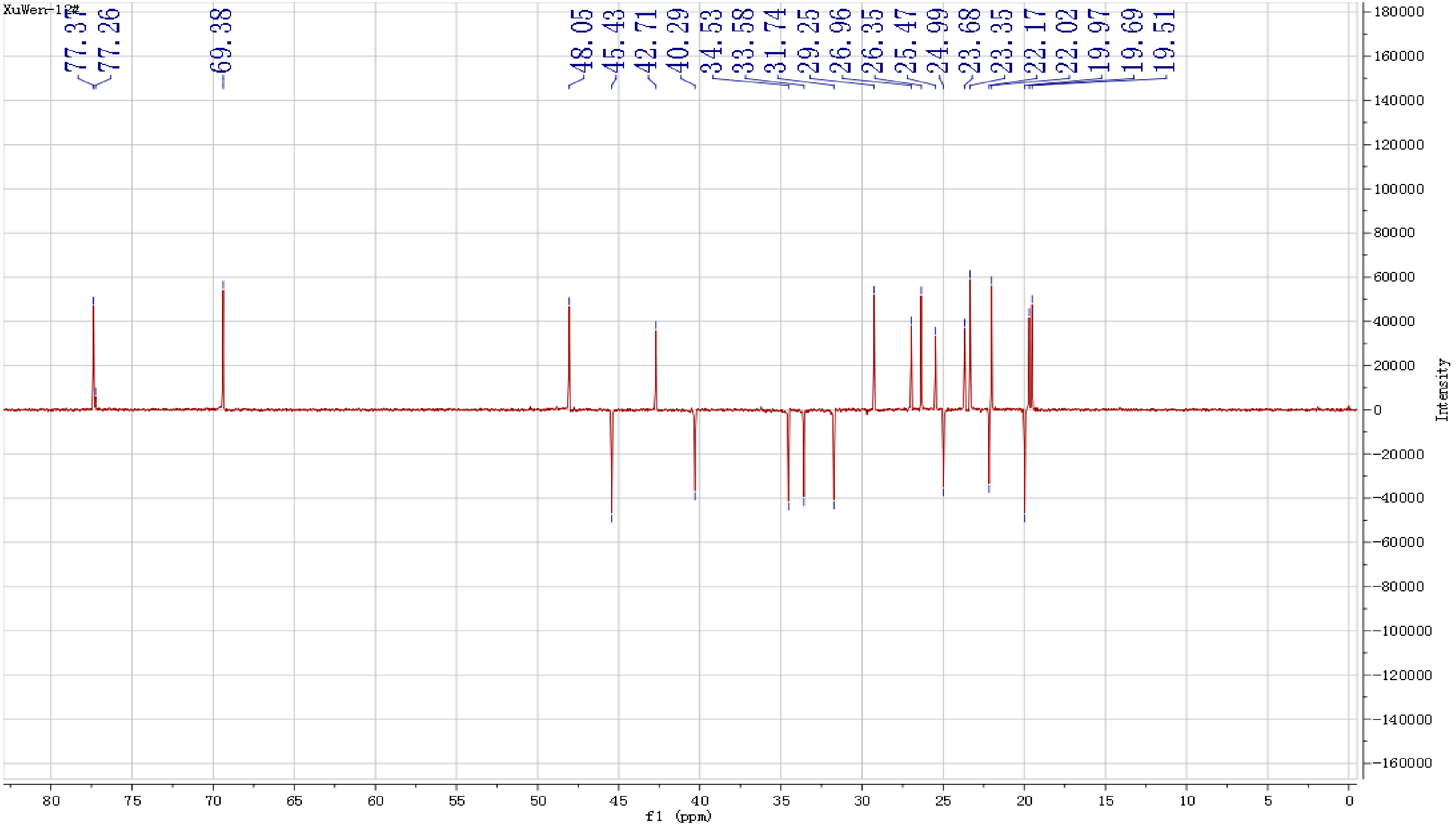

Figure S6. ^{13}C-NMR DEPT of 16-oxo-11-deoxy-alisol A.

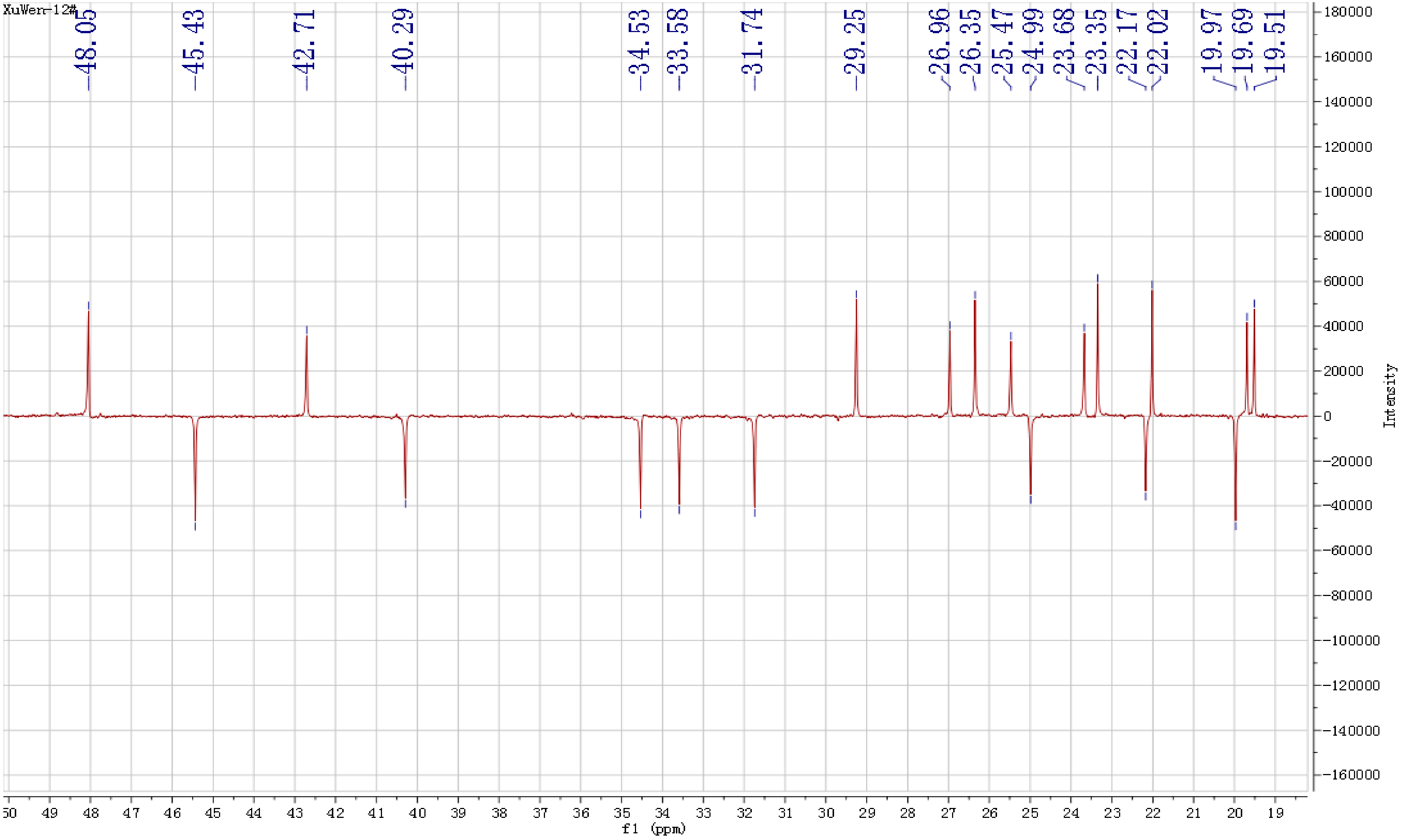

Figure S7. ^{13}C-NMR DEPT of 16-oxo-11-deoxy-alisol A—Expansion.
